# Adaptation of rhizobacterial and endophytic communities in *Citrus Grandis Exocarpium* to long-term organic and chemical fertilization

**DOI:** 10.3389/fmicb.2024.1461821

**Published:** 2024-10-21

**Authors:** Deyang Zhou, Kaiqing Yang, Yinhui Zhang, Cancan Liu, Ye He, Jialin Tan, Zhepu Ruan, Rongliang Qiu

**Affiliations:** ^1^Guangdong Laboratory for Lingnan Modern Agriculture, Guangdong Provincial Key Laboratory of Agricultural & Rural Pollution Abatement and Environmental Safety, College of Natural Resources and Environment, South China Agricultural University, Guangzhou, China; ^2^School of Environmental Science and Engineering, Sun Yat-sen University, Guangzhou, China

**Keywords:** *Citri Grandis Exocarpium*, fertilizer, root microbiome, microbial function, metabolome analysis

## Abstract

**Introduction:**

Organic fertilizers (OF) are crucial for enhancing soil quality and fostering plant growth, offering a more eco-friendly and enduring solution compared to chemical fertilizers (CF). However, few studies have systematically analyzed the effects of OF/CF on root microbiome of medicinal plants, especially in combination with active ingredients.

**Methods:**

In this study, we investigated the composition and function of bacteria and fungi in the rhizosphere or within the root of traditional Chinese medicinal plants, *Citri Grandis Exocarpium* (Huajuhong), which were treated with OF or CF over 1, 3, and 5 years (starting from 2018). Additionally, we conducted metabolome analysis to evaluate the effects of different fertilizers on the medicinal properties of Huajuhong.

**Results:**

The results indicated that extended fertilization could enhance the microbial population and function in plant roots. Notably, OF demonstrated a stronger influence on bacteria, whereas CF enhanced the cohesion of fungal networks and the number of fungal functional enzymes, and even potentially reduced the proliferation of harmful rhizosphere pathogens. By adopting distancebased redundancy analysis, we identified the key physicochemical characteristics that significantly influence the distribution of endophytes, particularly in the case of OF. In contrast, CF was found to exert a more pronounced impact on the composition of the rhizosphere microbiome. Although the application of OF resulted in a broader spectrum of compounds in Huajuhong peel, CF proved to be more efficacious in elevating the concentrations of flavonoids and polysaccharides in the fruit.

**Discussion:**

Consequently, the effects of long-term application of OF or CF on medicinal plants is different in many ways. This research provides a guide for OF/CF selection from the perspective of soil microecology and aids us to critically assess and understand the effects of both fertilizers on the soil environment, and promotes sustainable development of organic agriculture.

## 1 Introduction

Traditional Chinese medicines (TCMs) are gaining increasing recognition and acceptance globally for their significant contributions to the prevention and treatment of various global public health crises and complex diseases (Cai et al., 2004; Klayman, 1985). *Citri Grandis Exocarpium* (Huajuhong in Chinese), a valuable TCM with a centuries-old history, is utilized as an antitussive, expectorant, digestive aid, and for lowering blood sugar and lipid levels (Kong et al., 2020). Huajuhong is immature or nearly mature, dried exocarp of *Citrus grandis* “Tomentosa” or *Citrus grandis* (L.) Osbeck (Committee for the Pharmacopoeia of People’s Republic of China, 2015). Currently, in the context of circular agriculture, there is a growing trend towards using organic fertilizers (OF), such as the waste manure of livestock and poultry, to enhance the quality of perennial Chinese medicinal plants. Concurrently, the reliance on chemical fertilizers (CF) has significantly decreased. This trend is equally applicable to the cultivation of Huajuhong, with the consensus being that medicinal quality is higher when OF is used (Kong et al., 2020).

Fertilizer plays a vital role in enhancing crop yields and significantly influences agricultural productivity (Xu et al., 2024). In China, CF has been reported to contribute up to 56.81% to crop yield (Yang and Lin, 2019). Due to the rapid growth of extensive livestock and poultry farming, there is a substantial and concentrated accumulation of manure (Wang et al., 2022). The practice of returning breeding manure to agricultural land is a solution that addresses issues related to livestock and poultry breeding pollution, as well as agricultural non-point source pollution, promoting green development (Wang et al., 2021). This has brought OF back into focus which are environmental friendly and sustainable offer various advantages. However, OF also have drawbacks such as slower nutrient release compared to CF, potentially leading to slower plant growth. They can also be more costly and may not provide nutrients in the precise ratios required by plants (Spanoghe et al., 2020). Additionally, the nutrient content of OF can vary, posing challenges in accurately predicting and controlling nutrient availability for optimal plant growth (Shaji et al., 2021). Determining the optimal composition and ratio of OF remains a challenge that requires resolution, presenting an opportunity to draw insights from successful CF practices. Fertilizers also influence soil microbial diversity. OF, in addition to containing a large amount of organic matter and essential nutrients for crop growth, also contain a multitude of functional microorganisms. Numerous studies have reported that the gradual replacement of CF with OF has significantly promoted the diversity of soil microbial communities and soil enzyme activity (Bastida et al., 2021), thereby promoting plant growth, enhancing plants’ disease resistance and resistance to diseases, and improving the utilization rate of fertilizers (Wei et al., 2019). CF, when used excessively, may reduce soil microbial diversity, potentially decreasing soil fertility (Xu et al., 2020).

The root microbiome, often referred to as the “second genome” of plants, is crucial for plant growth and development (Bakker et al., 2013). Microbes play an important role in enhancing plant growth, such as nutrient supply (including nitrogen, phosphorus, potassium, sulfur, and micronutrients) and disease prevention (de Cássia Mesquita da Cunha et al., 2024). These beneficial activities predominantly take place in the rhizosphere—the narrow soil zone surrounding the roots—which serves as the primary site for nutrient exchange between plants and their surroundings (Ling et al., 2022; Philippot et al., 2013; Zuo et al., 2021). It’s also where most of the plant-associated microbes gather to access nutrients derived from the roots (Athul et al., 2022; Verma et al., 2022). Within these niches, microbes establish nonpathogenic relationships as endophytes within the roots, and form mutually beneficial associations like mycorrhizal fungi (Chen et al., 2024), while also potentially harboring harmful symbionts detrimental to plant health (Trivedi et al., 2020). These microbial interactions in the rhizosphere and within plant root tissue play a crucial role in regulating various aspects of plant life, including growth, physiology, yield, and resilience against both abiotic stresses (e.g., nutrient and water availability) and biotic stresses (Ruan et al., 2024).

Endophytes have the potential to be passed down from the parent host to its offspring (seeds) vertically, whereas the majority of bacterial endophytes are horizontally transmitted through the surrounding environment into the host (Frank et al., 2017; Li et al., 2023). Microorganisms on and around the plant surface can affect the survival and colonization of endophytes (Hardoim et al., 2008; Kandasamy and Kathirvel, 2023). In the early stage of invasion, microorganisms must adapt to the complex environment outside the host plant and overcome adverse factors to achieve invasion (Frank et al., 2017), so endophytes are considered to be a subgroup of rhizosphere microbiota. The assembly of endophytes in plants is driven by many factors, including soil type, host secretions, and artificial management (application of OF), and preferences for colonization in different ecological niches of plants (Dennis et al., 2010; Huang et al., 2023). The intricate relationship between plant roots, soil, and soil microorganisms forms a vibrant and resilient ecosystem. These components engage in a cycle of interaction and regulation. Through their exudates, plant roots foster certain microorganisms (Feng et al., 2023), which, in return, support the plant’s growth and development. This mutualistic relationship is instrumental in enhancing the soil’s ecological health, facilitating the plant’s nutrient absorption, and bolstering the host plant’s defenses against diseases (Dhanabalan et al., 2024). Notably, exploration into the rhizosphere microbiome and endophytes of medicinal plants is still in its infancy.

**Figure 1:**
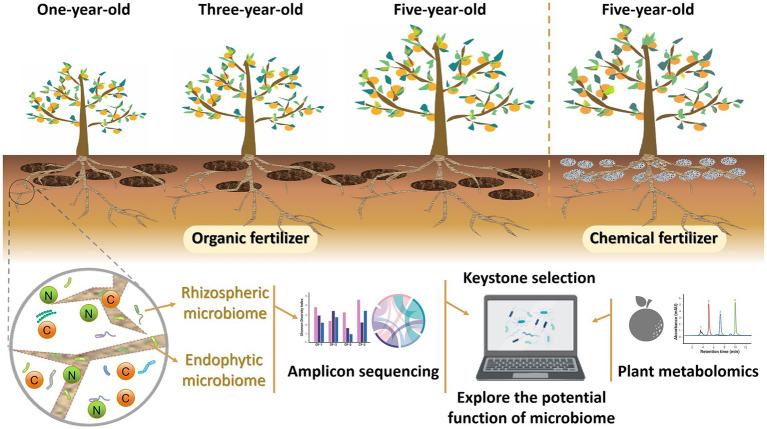
Schematic figure of the experimental design.

Current studies primarily concentrate on examining the impacts of OF on Chinese medicinal ingredients, with limited attention given to the effects of CF, particularly on Huajuhong. The comparison between the impact of CF and OF on medicinal ingredients remains insufficient. Whether the influence of CF on Huajuhong can be used as a reference for the application of OF is worth thinking about. In this study, we conducted a comprehensive analysis by screening soil samples collected from various years, different fertilizer applications, and different root regions. Through amplicon sequencing and non-target metabolomics analysis, we examined the microbiome composition, major enzyme types, and contents in both the rhizosphere and roots of the Huajuhong plant. Additionally, we investigated changes in fruit metabolites (Figure 1). Our objectives encompassed identifying: (1) variations in the structure and function of the root microbiome under long-term OF application compared to CF, (2) the impact of prolonged OF use on fruit metabolites in contrast to CF, and (3) strategies to leverage the benefits of CF for Huajuhong compared to OF. By delving into these data-driven studies and analyses, we aim to address whether CF outperform OF, pinpoint the key microbes or influencing mechanisms shaping Huajuhong quality during its growth, and furnish theoretical backing for advocating the substitution of CF with OF.

## 2 Materials and methods

### 2.1 Experimental design and sampling

The experiment has been carried out in Huajuhong Planting Base of Meihua, Zhongtong Town, Huazhou City, Guangdong Province, China since 2018 (21°29′–22°13′N, 110°20′–110°45′E). In February 2018, 10 Huajuhong seedlings were planted, with five receiving continuous application of OF and five receiving continuous application of CF under the same fertilization schedule and conditions. In February 2020 and February 2022, an additional five Huajuhong seedlings were planted every time and treated with OF. The OF consisted of chicken and pig manure, brown sugar (for fermentation), fish bone meal (to supplement calcium), peanut oil bran (for nitrogen and phosphorus supplementation), tobacco bone meal (for potassium supplementation and as a pesticide), and marine fish. This mixture undergone a 45-day composting process before application. Additionally, CF with a composition of 16:8:24 (N:P:K) is purchased directly as a commodity fertilizer.

In December 2022, rhizosphere soil and young roots from the 0–20 cm depth of the roots of the 1-year OF treated (OF-1), 3-year OF treated (OF-3), 5-year OF treated (OF-5), and 5-year CF treated (CF-5) Huajuhong plants were collected. Young root samples were collected with a length of 3 cm at the root tip. Each sample was collected by a five-point method, followed by sample mixing for a total of five replicates. Root samples and rhizospheric soil samples were collected for 16S rRNA gene high throughput sequencing as described by Edwards et al. (2015). The initial physicochemical properties of soil samples were measured by previously described methods.

Huajuhong fruits were harvested with similar size from five-year-old trees treated with CF or OF in July 2022. Following a standardized production process, the fresh fruits were briefly boiled in water for 1–3 min to halt enzymatic activity, transitioning the fruits color from green to yellow-green. Subsequently, the fruits were air-dried to room temperature, then transferred to a drying room where the temperature was maintained at 55–60°C for approximately 30–36 h until the moisture content reached below 12%. This process resulted in the production of Huajuhong medicinal fruit. The majority of the active components of the fruit are concentrated in the peel. To extract these components, the fuzzy peel on the surface of the fruit was carefully removed using a knife, and fruits samples from two treatments were made six repeats for metabolome detection.

### 2.2 DNA extraction and amplicon sequencing

Total microbial genomic DNA was extracted from a total of 20 soil samples and 20 root samples using the E.Z.N.A.^®^ soil DNA Kit (Omega Bio-tek, Norcross, GA, United States) according to manufacturer’s instructions. The quality and concentration of DNA were determined by 1.0% agarose gel electrophoresis and a NanoDrop2000 spectrophotometer (Thermo Scientific, United States).

PCR amplification of 16S rRNA gene was carried out in a 20 μL reaction mix using primer pairs 341F (5′-CCTAYGGGRB GCASCAG-3′) and 806R (5′-GGACTACNNGGGTATCTAAT-3′) for the hyper variable region V3–V4 (Xun et al., 2018), and the primers ITS1 (5′-CTTGGTCATTTAGAGGAAGTAA-3′) and ITS2 (5′-GCTGC GTTCTTCATCGATGC-3′) for the ITS region (Jogaiah et al., 2013). PCR products were purified using the PCR Clean-Up Kit (YuHua, Shanghai, China) according to manufacturer’s instructions and quantified using the Qubit 4.0 (Thermo Fisher Scientific, United States). Amplicons were pooled in equimolar concentrations and sequenced using the Illumina MiSeq PE 250 platform (Illumina, San Diego, United States) by Majorbio Bio-Pharm Technology Co., Ltd. (Shanghai, China). After demultiplexing, the obtained sequence was subjected to quality filtering using fastp (0.19.6) (Magoč and Salzberg, 2011) and merged using FLASH (v1.2.11). Then the high-quality sequences were de-noised using DADA2 (Chen et al., 2018) plugin in the Qiime2 (version 2020.2) pipeline with recommended parameters (Bolyen et al., 2019), which obtained single nucleotide resolution based on error profiles within samples. Read pairs from each sample were merged and screened with a maximal expected error of 0.2 and a minimum length of 250 base pairs (bp). DADA2 denoised sequences are usually called amplicon sequence variants (ASVs). Following the merging of paired reads and chimera filtering, the taxonomic classification of each ASV was determined using the Silva (SSU132) 16S rRNA database and Unite (Release 7.2 https://unite.ut.ee/index.php) with a confidence threshold of 70%.

### 2.3 Statistical analysis

All statistical analyses were performed using GraphPad Prism Software (version 8.0) and the R package (v4.0.3). Student’s *t*-test was used to compare the group means in the statistical analyses. Statistically significant differences were indicated as follows: ^*^p < 0.05 and ^**^p < 0.01.

Biodiversity indices such as species diversity, richness, and evenness were calculated using functions in Vegan (Ruan et al., 2024). The Chao diversity index was used to estimate microbial diversity for each group. We used principal coordinates analysis (PCoA) with Bray–Curtis distances to ordinate the samples based on their dissimilarity using the “metaMDS” function in Vegan. The microbiome data graphs were generated using the “ggplot2” (v3.3.0) and “VennDiagram” (v1.6.20) packages in R.

All molecular ecological networks were constructed on the basis of Pearson correlations of log-transformed ASV abundances, followed by an RMT-based approach that determines the correlation cut-off threshold in an automatic fashion. The ASVs with the top 500 average relative abundance at each time point in each treatment were selected, and their co-occurrence networks were computed separately using the Molecular Ecological Network Analysis Pipeline (MENAP)^1^ (Deng et al., 2012). To ensure the reliability of correlation calculation, only ASVs present in 5 of the 5 samples were included for correlation calculation. We used the same cut-off value (0.88) of correlation coefficient for all networks. The topological roles of ASVs (nodes in the network) was assessed by determining their Zi (connections within modules) and Pi (connections between modules) values using the rnetcarto package in R (v4.0.3). The importance of each node was categorized into four sections based on Olesen’s et al. (2007) methodology. Network visualization was carried out using Gephi (v0.9.1) (Bastian et al., 2009). PICRUSt2 (version 2.3.0b) software was utilized to predict the functional gene products (Enzyme Commission numbers, EC numbers) based on the taxonomic information obtained from the 16S rRNA sequencing database (Douglas et al., 2020). Distance-based redundancy analysis (dB-RDA) was used to assess the potential contribution of fertilizer composition to microbiome in different treatments in R.

### 2.4 Non-target metabolomics analysis of Huajuhong fruits

Six parallel replicates for each treatment group were used in our non-targeted metabolomics investigation of five-year-old OF and CF Huajuhong fruits. Fifty milligrams solid sample was first added to a 2 mL centrifuge tube and a 6 mm diameter grinding bead was added. Four hundred microliters of extraction solution [methanol: water = 4:1 (v:v)] containing 0.02 mg/mL of internal standard (L-2-chlorophenylalanine) was used for metabolites extraction. Samples were ground by the Wonbio-96c (Shanghai Wonbio Biotechnology Co., Ltd.) frozen tissue grinder for 6 min (−10°C, 50 Hz), followed by low-temperature ultrasonic extraction for 30 min (5°C, 40 kHz). The samples were left at −20°C for 30 min, centrifuged for 15 min (4°C, 13,000 g), and the supernatant was transferred to the injection vial for LC-MS/MS analysis. As part of the system conditioning and quality control procedures, a pooled quality control (QC) sample was created by combining equal volumes of all individual samples. These QC samples underwent disposal and testing in the same manner as the analytical samples. This practice aided in representing the entire sample set and was utilized for regular monitoring of analysis stability by injecting it at specified intervals. The details of (UHPLC-MS) analysis and data analysis are described in Supplementary Methods. When comparing the metabolomes of CF and OF to map the volcano plot, the initial step involves assessing overall differences between the two groups through principal component analysis (PCA) and partial least squares discriminant analysis (PLSDA). Subsequently, differential metabolites are identified by analyzing the variable importance in projection (VIP) values from the orthogonal partial least squares discriminant analysis (OPLSDA), complemented by univariate analysis focusing on fold change and *p*-value, followed by the creation of a volcano plot to visually represent these data. It is important to note that if the OPLSDA model is overfitted, the VIP values from PLSDA should be utilized instead. This approach ensures a comprehensive evaluation of metabolite differences influenced by the application of CF and OF treatments.

## 3 Results

### 3.1 Fertilizers promote microbial diversity over time

**Figure 2:**
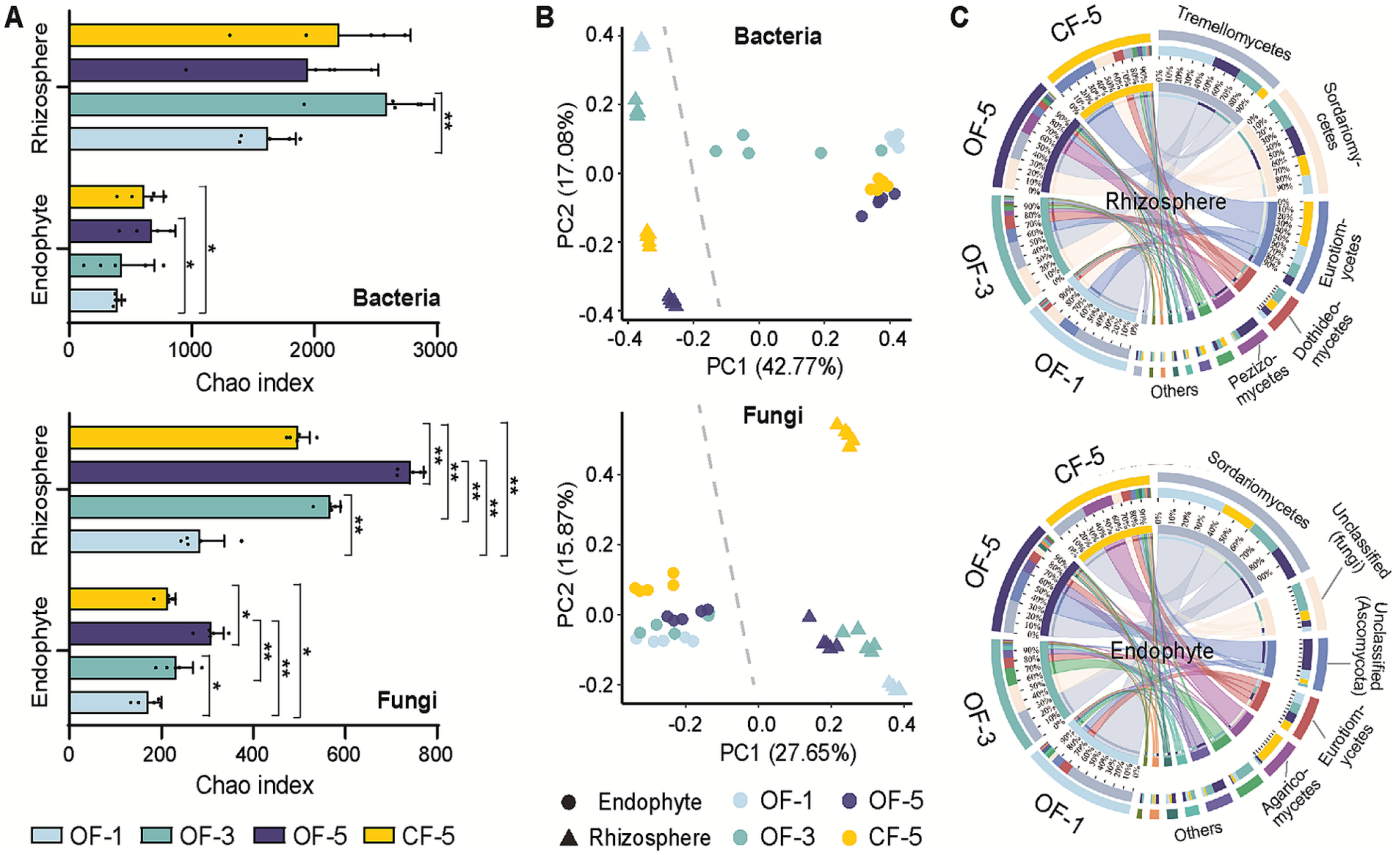
Diversity analysis of microbial communities. (A) α-diversity analysis (using the Chao index) for bacteria and fungi. The data are presented as mean values ± SD (*n* = 5 biological independent replicates). The significance of differences was assessed using a two-sided student’s *t*-test (^**^p < 0.01 and ^*^p < 0.05). (B) Principal coordinates analysis (PCoA) plots of β-diversity (Bray–Cutis dissimilarity). (C) Taxonomic composition of the fungal microbiome at the phylum level. OF-1, one-year tree treated by organic fertilizer; OF-3, three-year tree treated by organic fertilizer; OF-5, five-year tree treated by organic fertilizer; CF-5, five-year tree treated by chemical fertilizer.

To investigate the temporal evolution of microbial communities under various fertilization treatments, the alterations in bacterial and fungal microbial communities were examined at 1-year, 3-year, and 5-year. A histogram of Chao index was employed to illustrate the species abundance within each treatment, revealing that both OF and CF enhanced the diversity of rhizosphere microbial and endophytic communities. Fertilizers promoted the abundance of bacteria significantly more than that of fungi. Notably, OF exhibited a greater propensity to enhance the diversity of bacterial and fungal species compared to CF. The microbial diversity of CF-5 was always lower than that of the 3- and 5-year OF treatments (Figure 2A).

The analysis of β-diversity also confirmed differences in the composition between rhizosphere and endophyte microbiome regardless of the treatments (Figure 2B). Compared with OF, CF had a greater effect on the structure of fungi, especially on rhizosphere. The principal coordinates analysis (PCoA) diagram revealed that the variances between rhizosphere bacteria and bacterial endophytes remained relatively stable over time. In contrast, the β-diversity distance between rhizosphere fungi and fungal endophytes exhibited a gradual decrease over time, suggesting that endophytes were influenced by rhizosphere fungi and displayed a tendency towards convergence.

In terms of species composition, there were variations observed in the samples from year to year and across different treatments. For example, *Eurotiomycetes* exhibited a notably high proportion in the rhizosphere of CF-5, while the highest abundance of *Sordariomucetes* was observed in OF-3 and OF-5 when considering bacteria. In the case of endophytes, the species that held dominance in the initial year did not maintain their dominance in the third and fifth years. It is worth noting that the abundance of *Agaricomycetes* treated with CF was higher than the abundances corresponding to the other groups (Figure 2C; Supplementary Figure S1). *Agaricomycetes* are large fungi with crucial symbiotic relationships with plants. They form mycorrhizal associations that facilitate nutrient exchange, enhancing plant growth and health. Their presence indicates soil health and biodiversity, and they play a vital role in ecosystem sustainability through their contributions to nutrient cycling and organic matter decomposition (Lutzoni et al., 2018). This pattern of substantial variation in species composition was also evident in bacteria, with distinct differences observed in the composition of endophytic and rhizosphere bacteria (Supplementary Figure S2).

### 3.2 Microbial network properties under different fertilization conditions

**Figure 3:**
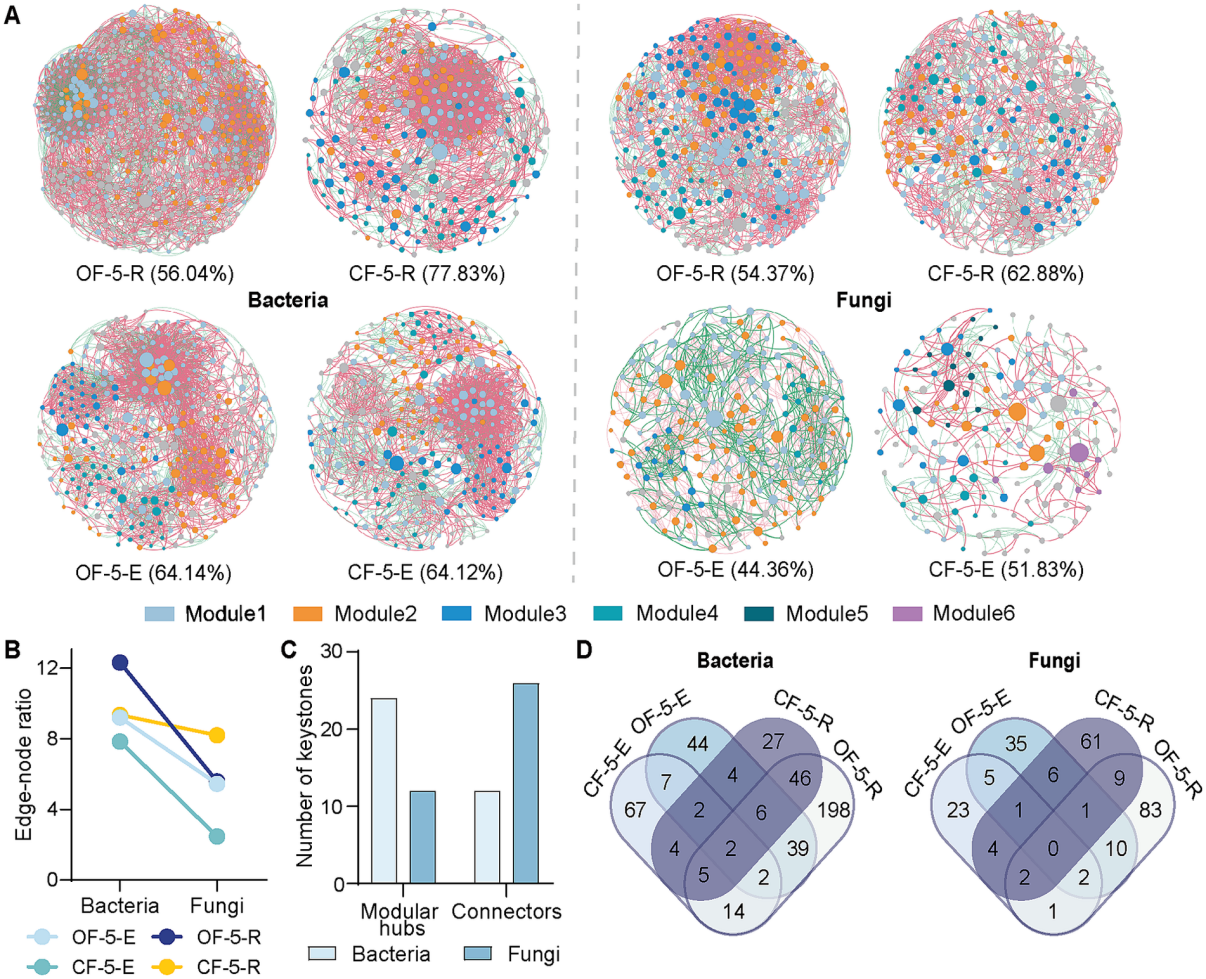
Microbial co-occurrence network analysis of five-year-Huajuhong. (A) Co-occurrence networks of microbiomes associated with CF and OF treatments from fifth year trees. The percentage values represent the positive correlation index of the networks. (B) Edge-note ratio of bacteria and fungi of the network parameters. (C) Number of keystone species through co-occurrence network analysis based on Zi-Pi values. Species with Zi <2.5 and Pi >0.62 are connectors, and Zi >2.5 and Pi <0.62 are modular hubs. (D) Venn diagram based on the network module with the highest number of microorganisms in each sample. OF-5, five-year tree treated by organic fertilizer; CF-5, five-year tree treated by chemical fertilizer (*n* = 5 biological independent replicates). E, endophyte; R, rhizosphere.

We constructed co-occurrence networks based on Spearman correlations to illustrate the influence of long-term application of fertilizers on microbial community structure (Figure 3A). Our findings indicated distinct co-occurrence patterns in the bacterial and fungal communities across various fertilizer treatments. When applying the same threshold value, the extended use of OF resulted in a more tightly connected microbial co-occurrence network. However, in comparison to OF treatment, the positive correlation ratio in the CF network was higher, particularly with a positive correlation ratio of 77.83% observed in bacterial rhizosphere group. Interestingly, co-occurrence network properties of fungi were always notably lower in the OF group compared to the CF treatment group, indicating a greater impact of CF on the fungal network structure (Figure 3B; Supplementary Figure S3).

The network’s within-module degree (Zi) and among-module degree (Pi) of nodes were simultaneously determined using the greedy module optimal algorithm. Nodes with Zi <2.5 and Pi <0.62 are classified as peripherals, while those with Zi <2.5 and Pi >0.62 are connectors. Nodes with Zi >2.5 and Pi <0.62 are considered modular hubs, and nodes with Zi >2.5 and Pi >0.62 are network hubs (Ling et al., 2022). In a community, connectors, modular hubs, and network hubs can all be regarded as potential keystone taxa of the community. Our results indicated that keystones from CF or OF groups played a role in promoting modular hubs and connectors within the network. This suggested that fungi facilitated the connection and interaction between modules, while bacteria primarily contributed to interactions within modules (Figure 3C; Supplementary Figure S4).

The Venn diagram illustrated the species information present in the largest module of the network diagram for each treatment group. Analysis of the diagram revealed that there was a high degree of similarity between bacterial endophytes and rhizosphere bacteria in the OF group, with minimal impact of CF treatment on endophytes. Conversely, in the fungal network, there was little similarity observed between endophytes and rhizosphere fungi across different groups (Figure 3D). We also analyzed the degree values of top10 genus/family with significant differences between CF and OF treated groups in the co-occurrence network (Supplementary Figures S5, S6). These top 10 microorganisms in the network, no matter in CF or OF group, their network degrees were relatively consistent indicating similar important role in the network. For example, *Burkholderia* had a high degree in the OF network as well as in the CF network, while *Dyella* had a low degree of network in both groups. The situation was similar in both fungi and bacteria.

### 3.3 Contribution of soil properties to the variation of the microbial community

The soil basic chemical properties are shown in Supplementary Table S1. The long-term application of fertilizer led to a decrease in pH value, and the acidification caused by CF was relatively stronger. The contents of ammonium, nitrate and available-N in OF-treated soil were relatively low, while the contents in CF-treated soil were higher and more stable.

**Figure 4:**
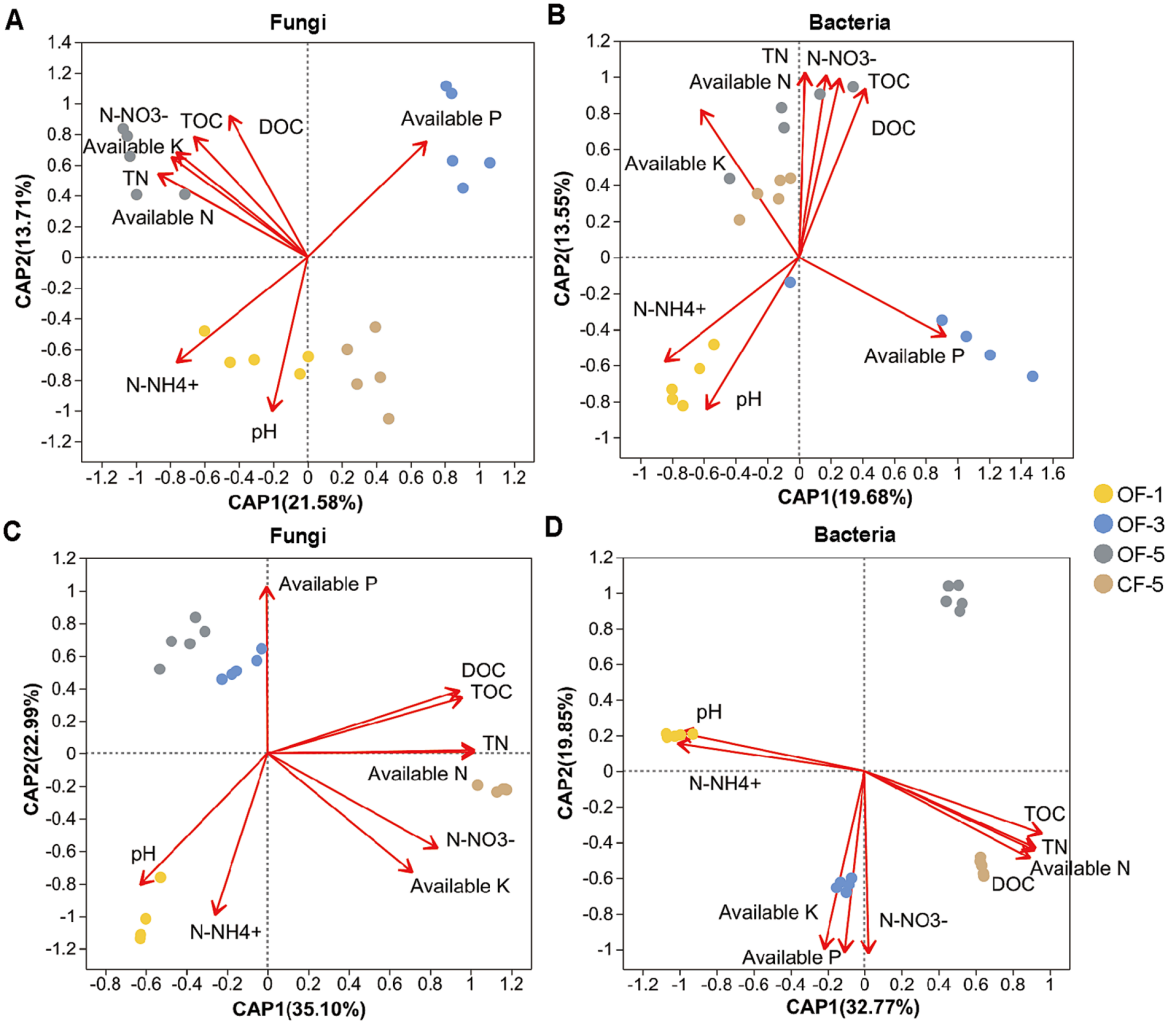
Distance-based redundancy analysis (dB-RDA) demonstrates shifts in the microbial community’s pattern at the genus level as affected by soil properties. (A) Fungal endophytes. (B) Bacterial endophytes. (C) Rhizosphere fungi. (D) Rhizosphere bacteria. OF-1, one-year tree treated by organic fertilizer; OF-3, three-year tree treated by organic fertilizer; OF-5, five-year tree treated by organic fertilizer; CF-5, five-year tree treated by chemical fertilizer (*n* = 5 biological independent replicates). TOC, total organic carbon; TN, total nitrogen; DOC, dissolved organic carbon.

RDA was used to illustrate the relationship between the soil properties and the microbial diversity. The explanatory variables, including nitrate, available nitrogen, total nitrogen (TN), total organic carbon (TOC), available potassium (K), and dissolved organic carbon (DOC), were the main factors influencing endophytes in OF-5 group and bacterial endophytes in CF-5 group (Figures 4A,B). As for rhizosphere, the effects of CF on soil factors and microbiome were more obvious than that of OF, where all soil factors except ammonium and pH were the main factors affecting CF-treated rhizosphere microorganisms, whether it was for bacteria or fungi (Figures 4C,D).

### 3.4 Microbial functional alteration under different fertilization treatments

**Figure 5:**
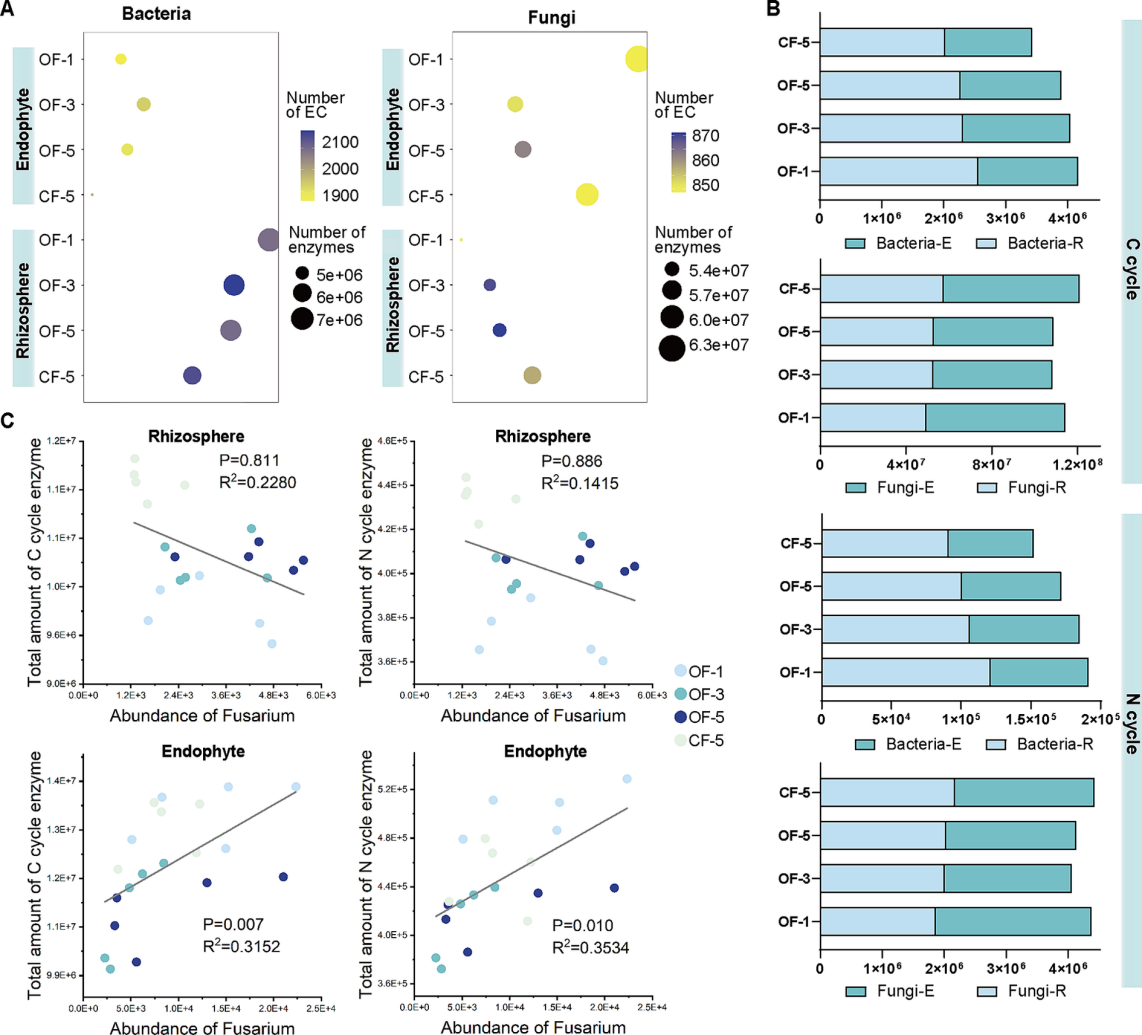
Functional analysis of bacterial and fungal microbiome. (A) Bubble diagrams based on the types and abundance of bacterial (left)/fungal (right) metabolic enzymes and ECs. (B) Number of C/N cycle-related metabolic enzyme coding genes among different samples. (C) The number of pathogenic fungi *Fusarium* decreased with the number of enzyme coding genes associated with the C/N cycle of rhizosphere microbiome, and increased with the number of enzyme coding genes associated with the C/N cycle of endophytes. Linear regression line is indicated by the grey line. *p*-values are two-sided. OF-1, one-year tree treated by organic fertilizer; OF-3, three-year tree treated by organic fertilizer; OF-5, five-year tree treated by organic fertilizer; CF-5, five-year tree treated by chemical fertilizer (*n* = 5 biological independent replicates). E, Endophyte; R, rhizosphere.

OF and CF also had a great impact on microbial function. After long-term application of OF, the enzyme content of both endophytic and rhizosphere microbiome showed a decreasing trend (except rhizosphere fungi). CF promoted both the total enzyme amount and the number of ECs of the endophytic and rhizosphere fungi, which was much higher than the OF-treated group (Figure 5A).

The abundance of carbon (C) and nitrogen (N) in the environment were the main factors affecting microbial activities, so we focused on analyzing the enzyme coding genes related to C cycle and N cycle in the different treatments (Figure 5B). In the C cycle, the enzyme coding genes for both bacteria and fungi in the OF group gradually decreased over time. However, applying CF decreased the enzyme coding genes involved in C cycle of bacteria which was lower than OF-5, but increased the abundance of related genes of fungi. The abundance of enzyme coding genes related to N cycle showed a similar pattern. Additionally, the abundance of enzyme coding genes for rhizospheric bacteria involved in C and N cycle was higher in all treatments than that of endophytic bacteria; whereas it was opposite for fungi.

*Fusarium* was the most abundant fungi in all samples, and many species of this genus were pathogens. In order to understand the interaction between soil microbial function and *Fusarium*, we analyzed the correlation between the abundance of the enzyme coding genes in C cycle and N cycle and the abundance of *Fusarium* (Figure 5C). In the rhizosphere, the number of enzyme coding genes related to C cycle or N cycle was negatively correlated with the abundance of *Fusarium*, while in the endophyte, there was a positive correlation. The abundance of the genes of fungi was higher than that of bacteria, especially in the CF treated group, indicating that it was more likely to lead to the growth of pathogens in plants (Figure 5B). However, it is interesting to note that the abundance of *Fusarium* in the CF group was lower than that in OF groups, especially in the rhizosphere.

### 3.5 Metabolomics analysis of Huajuhong fruits

**Figure 6:**
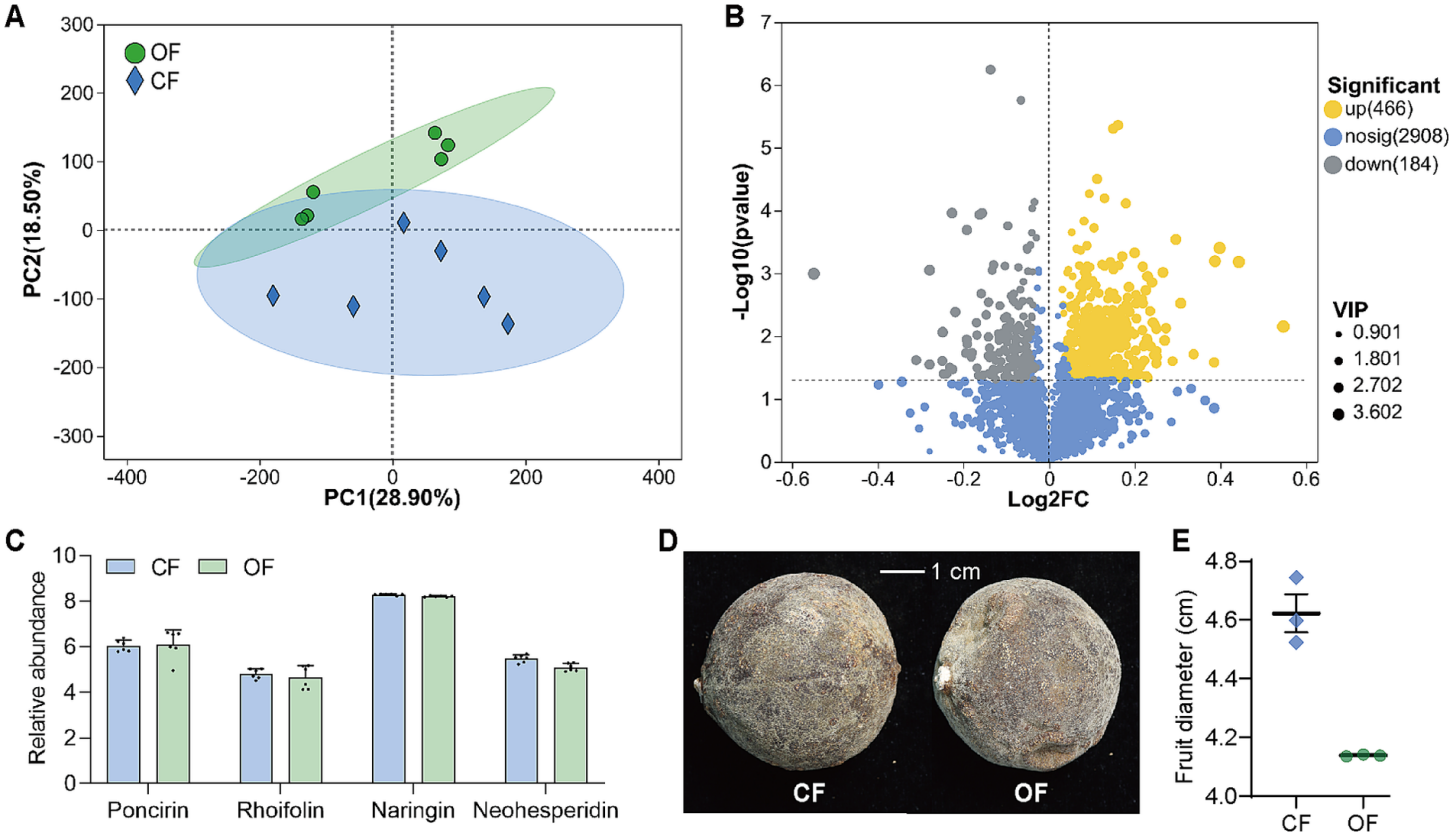
Plant non-target metabolomics. (A) Principal component analysis (PCA) of citrus peel metabolome (*n* = 6 biological independent replicates). (B) Volcano plot of significant difference analysis of active ingredients. Yellow dots, substances were significantly upregulated in OF. Gray dots, substances were significantly upregulated in CF. The VIP value represents the impact strength of the corresponding metabolite’s group differences in the model’s sample classification discrimination among groups. It is generally considered that metabolites with VIP ≥1 are significantly different. (C) Relative abundance of the four main medicinal flavonoid substances. (D) Photos of Huajuhong fruits. (E) Diameters of Huajuhong fruits (*n* = 3 biological independent replicates). OF, organic fertilizer; CF, chemical fertilizer. The data are presented as mean values ± SD.

The effects of OF and CF on fruit metabolism were relatively significant in principal component analysis (PCA) of citrus peel metabolome (Figure 6A), which included 294 carbohydrate-polysaccharide substances, 616 flavonoids, 207 coumarins, etc. 3,558 substances were detected, and 466 were significantly upregulated in the OF treated Huajuhong peel and 184 substances were significantly upregulated in CF treated Huajuhong peels (Figure 6B; Supplementary Figure S8). Flavonoids, polysaccharides, monoterpenes, coumarins and other active ingredients with pharmacological effects were contained in Huajuhong. However, there was no significant difference in the abundance of these substances in different groups (Supplementary Figure S6). Meanwhile, there was also no significant difference in the abundance of the four main medicinal flavonoid substances (poncirin, rhoifolin, naringin, and neohesperidin) (Figure 6C). The average diameter of fruits treated with OF was 4.14 cm, and that treated with CF was 4.62 cm (Figure 6D). The results showed that the application of OF had no significant effect on the overall yield of the main substances exerting the medicinal effect. However, since the fruits in OF group were smaller, OF did regulate the significant secretion of more metabolites.

## 4 Discussion

### 4.1 Differences in root microbiome structure and function under extended OF vs. CF application

Soil physical and chemical properties are considered to be one of the key factors affecting microbial composition (Philippot et al., 2013). In this study, although the soil chemical properties were different in different planting years, the diversity analysis showed that the planting years was the main factor affecting the microbial community structure. Microbial diversity in the rhizosphere or endophyte gradually increased over time, especially for OF groups (Figure 2). *Proteobacteria*, *Actinobacteria*, *Chloroflexi*, and *Firmicutes* were bacteria with high abundance in the rhizosphere, and their relative abundance changed differently under different planting years, which is consistent with previous results that root-associated bacterial communities are predominantly composed of members from the phyla *Actinobacteria*, *Bacteroidetes*, *Firmicutes*, and *Proteobacteria* (Bulgarelli et al., 2013). Similarly, bacterial community function showed a “time-decline” pattern in both sites, which also confirmed the similar results. Interestingly, the opposite was true for fungi, where functional diversity firstly decreased and then increased over time (Figure 5). Previous studies have shown that the composition of many plant rhizosphere bacteria and fungi (e.g., *Arabidopsis thaliana*, *Astragalus mongholicus*, sugarcane, pea, wheat, and soybeans) (Leite et al., 2021; Li et al., 2023; Lundberg et al., 2012) varies with the stage of plant development. It has been confirmed that different root microorganisms will be formed due to the differences in root exudations of plants at different developmental stages. As for Huajuhong, it takes at least 5 years to begin the first round of fruit production (Committee for the Pharmacopoeia of People’s Republic of China, 2015), so the rhizosphere microbes would be related to planting years (tree age).

The type of fertilizers was another important factor. The overall trend for both bacterial and fungal abundance was to increase with the duration of OF application. Long-term application of CF only promoted abundance of rhizosphere bacteria, but inhibited microbial diversity for fungi and endophytic bacteria. Studies have shown that long-term application of CF increases the effective phosphorus content in the rhizosphere environment. This enhancement not only stabilizes the soil bacterial community but also boosts its overall abundance (Xiao et al., 2024). This is consistent with the reported rhizosphere of non-medicinal plants (Coleman-Derr et al., 2016; Ling et al., 2022). There was a great distance of β-diversity between the CF and OF groups, especially in the rhizosphere. Although there were significant differences in rhizosphere microorganisms and endophytes, the effects of OF and CF on microbial abundance and function in the two sites were consistent, especially the enrichment of key species. Previous studies have shown that inoculation of *Burkholderia* in citrus roots can promote the metabolic reactions related to carbohydrates and amino acids in the microbiome (Wang et al., 2023). In this study, we also found that *Burkholderia* was always the most dominant strain in both rhizosphere and roots of Huajuhong, and the abundance in the OF group was significantly higher than that in the CF group (Supplementary Figure S6), even with a high degree in co-occurrence networks, indicating *Burkholderia* could be used as a biofertilizer combined with OF to promote plant growth (Supplementary Figure S5).

Applying OF over an extended period can raise the soil’s organic carbon content and supply enough substrate for microbial growth (Chen et al., 2023). This effect is stable and long-lasting, and will get stronger with the longer the application (Zhang et al., 2010). In our study, the bacteria in the root were affected by CF/OF chemical properties in the same way, while CF/OF had opposite effects on rhizosphere bacteria and fungi. Most endophytes came from the rhizosphere, so the effect of accurate application of fertilizer on endophytes may not be ignored, especially the prevention and control of pathogens.

### 4.2 Effects of CF or OF on soil pathogens and probiotics

In the rhizosphere, plants are constantly exposed to a diverse array of microbial populations, including commensals, pathogens, and symbionts. Plant pathogens and nitrogen-fixing bacteria, such as *Rhizobium* sp., are particularly abundant in the rhizosphere due to their reliance on organic matter supplied by the plant host for reproduction and function (Supplementary Figure S6). Despite the dynamic nature of the rhizosphere and the rapid evolution of its microbiome over time and space, there is growing evidence to suggest that plants actively shape the rhizosphere microbiome to their advantage and effectively utilize the functional capabilities of these microbes (Bakker et al., 2013; Ling et al., 2022).

*Fusarium* is one of the most destructive agricultural pathogens, causing great damage to a wide variety of plants (Guo et al., 2022; Li et al., 2022; Zhou et al., 2022). Although no studies have reported the effect of *Fusarium* on Huajuhong, in this study, a high abundance of *Fusarium* was detected in the roots of Huajuhong. In the rhizosphere, the application of OF can lead to a decrease in the abundance of *Fusarium* due to the introduction of many non-indigenous microorganisms and the increased availability of easily degradable organic carbon sources that stimulate the growth of other microbial groups (Wen T. et al., 2020). Inside roots, however, the presence of OF provides a rich growth substrate for *Fusarium*, and since the microbial diversity inside the roots is not as abundant as in the rhizosphere, *Fusarium* becomes dominant (Shu et al., 2022; Wen Y. C. et al., 2020). Under the influence of low C/N cycle genes in the rhizosphere of OF groups, the abundance of *Fusarium* was negatively correlated with the expression of C/N cycle genes, while in the root, there was a positive correlation (Figure 5C). Interestingly, the CF groups always had the lowest *Fusarium* abundance. This is consistent with the results reported in previous studies that long-term application of chemical nitrogen and phosphorus fertilizer combined with organic improvement provided a secure disease management strategy for suppressing *Fusarium* root rot of soybean (Liu et al., 2021).

In addition to *Fusarium*, the application of OF or CF also had different effects on other soil pathogens and probiotics. We selected the top 10 species with the highest abundance in each group, and the number of probiotics in the rhizosphere decreased with time. Almost all top 10 rhizosphere bacteria were probiotics, while rhizosphere fungi accounted for a higher proportion of pathogens in OF groups than in CF group (Supplementary Tables S4, S5). The situation in Huajuhong root was different, the number of fungal pathogens first decreased with time and then raised again (Supplementary Tables S2, S3). Although the effect of CF on fungi was more significant, for probiotic bacteria, CF could promote their abundance more than OF, especially in the root. It is not surprising that plant pathogens are more prevalent in the rhizosphere compared to the surrounding bulk soil (Ling et al., 2022), which may be due to the following reasons: (1) the number of pathogenic fungi that are classified as soilborne is limited, likely due to the inability of non-spore forming fungi to thrive in bulk soils for extended periods of time (Raaijmakers et al., 2009); (2) the majority of plant pathogens exhibit saprophytic growth in the rhizosphere, deriving essential energy from the roots (Larsen et al., 2015); (3) some pathogens, whether they are potential symbionts or covert pathogens, show a preference for colonizing the rhizosphere (Hannula et al., 2021; Ma et al., 2020). The abundance and variety of harmful and beneficial microorganisms play a crucial role in determining the outcomes of microbial interactions in the rhizosphere. However, determining the optimal conditions for plant health and striking a balance between defense against pathogens and promotion of beneficial bacteria remains a challenging task. Therefore, the application of OF alone would lead to an increase in the number of pathogens, which was also consistent with previous studies (Pu et al., 2022), and it was recommended to supplement the application of CF or microbial OF, providing reference for the prevention and control of other microbial diseases of Huajuhong.

### 4.3 Optimizing OF application of Huajuhong from CF benefits

The key to the modernization of Chinese medicine is to ensure the stable chemical composition of Chinese medicine. Huajuhong is a famous herbal for resolving phlegm, suppressing cough and calming panting, and has been paid more and more attention. However, the cultivation methods of saffron have not been uniform, some use CF, and some use OF combined with CF. A series of studies have found that the application of OF can improve the appearance, color and odor of medicinal plants. The content of phenols, alkaloids, flavonoids and other components is significantly higher than that of no fertilization or CF application (Obidola et al., 2019; Pacheco et al., 2021). The results of this study are also consistent. Although the content of total flavonoids and naringin, the index of Chinese medicine to develop high quality products of Huajuhong (Li et al., 2014), was not significantly different between the OF and CF groups. There were more types and abundance of metabolites in Huajuhong fruits treated with OF than that treated with CF (Supplementary Figure S8). Therefore, it was beneficial to cultivate Huajuhong with OF.

The significant discharge of manure from the breeding industry and the excessive use of fertilizers in the planting sector exacerbate agricultural non-point source pollution, placing considerable strain on rural ecological management and the sustainable development of agriculture. The effective utilization of animal manure has emerged as a pressing issue in the sustainable advancement of animal husbandry. Implementing a green breeding cycle model that utilizes livestock and poultry manure to produce OF represents a crucial step towards promoting environmentally friendly, low-carbon agricultural practices and enhancing rural ecological conditions (Jin et al., 2021; Zhu et al., 2022). In this study, diverging from the traditional practice of utilizing livestock and poultry manure as raw materials for OF, we capitalized on the proximity to the sea to incorporate a range of agricultural wastes, including fish bones, marine fish, and tobacco residues, and the effect of OF was comparable to that of CF.

In certain OF, such as manure, the C/N ratio has been identified as a crucial factor influencing the variability in nitrogen mineralization from the initial OF (Qian and Schoenau, 2002). Numerous experiments have shown that the nitrogen released from OF may not be adequate for achieving economic production. Therefore, in addition to supplementing CF, the focus of future research lies in enhancing the efficient mineralization of organic nitrogen in OF through the activities of soil microorganisms. However, for Huajuhong, a perennial medicinal plant, the slow-release properties of OF were not as pronounced as they were for other crops. Furthermore, OF offers various advantages beyond mere nutritional benefits, with positive impacts on key soil characteristics such as water-holding capacity, cation exchange capacity, and microbial activity. Studies on OF in medicinal plants primarily examine the overall effects on yields, with less emphasis on the specific effects of individual nutrients. The composition of manure was complex, so it is necessary to carry out specific effects and risk analysis of the individual substances.

Plants exhibit varying nutrient requirements at different growth stages, leading to inconsistencies in the composition of aggregated microorganisms (Zhang et al., 2024). Variations in the species composition of inter-vegetative microorganisms during distinct developmental phases of Huajuhong not only impact soil metabolism but also indicate the plant’s adjustment to changing nutrient needs (Sun et al., 2022). To address the evolving requirements of Huajuhong across different growth stages, the application of OF alone or in conjunction with CF can enhance soil fertility and positively influence soil nutrient cycling (Zhang et al., 2022). Furthermore, apart from the diverse fertilizer demands at various growth stages, the plant rhizosphere microbial community becomes more responsive to soil environmental changes and may enhance soil carbon and nitrogen cycling following prolonged OF application (Zhao et al., 2023). Since the effects of chemical and organic fertilizers on microbial communities differed, particularly in their potential influence on pathogenic microbes. Based on the results of this experiment, it was not necessary to exclusively apply OF. When there was an increase in the number of pathogenic bacteria, CF could be utilized as a regulatory measure. This necessitates growers to exercise precise control over fertilizer application to meet the plant’s immediate requirements while mitigating the adverse effects of nutrient accumulation.

## 5 Conclusion

Given the pressing need to address agricultural non-point source pollution, our research offered insights into the judicious use of OF on TCM. Furthermore, our findings addressed the question posed in the title, revealing that OF was not inherently superior to CF and may lead to an increase in pathogenic fungi abundance in Huajuhong. In conclusion, while emphasizing the utilization of agricultural byproducts like livestock manure, a balanced approach incorporating various fertilizers, including chemical options, was recommended. With the increasing focus on the quality of cultivated medicinal plants, our findings have not only contributed to improving the quality of medicinal materials but have also endorsed the adoption of integrated planting and breeding techniques within a circular agricultural system. This approach promoted resource recovery and the environmentally safe disposal of waste materials generated by the planting and breeding industries. Our research provides both theoretical insights and practical support for the advancement of sustainable agricultural practices. With the advancement of integrated crop-livestock farming, organic fertilizers are gaining more attention for their future use. Besides the need to carefully combine them with inorganic fertilizers, it is crucial to take a holistic approach in considering the impact of harmful microorganisms, heavy metals, and organic pollutants found in organic fertilizers on the plant microbiome, which is referred to as a second genome of plant.

## Data Availability

All amplicon sequencing data have been deposited in the NCBI Sequence Read Archive (SRA) under the accession number PRJNA1152228 (for bacteria) and PRJNA1152714 (for fungi).

## Footnotes

^1^http://ieg2.ou.edu/MENA

## References

[ref1] Athul P. P. Patra R. K. Sethi D. Panda N. Mukhi S. K. Padhan K.. (2022). Efficient native strains of rhizobia improved nodulation and productivity of French bean (*Phaseolus vulgaris* L.) under rainfed condition. Front. Plant Sci. 13:1048696. doi: 10.3389/fpls.2022.1048696, PMID: 36589118 PMC9797659

[ref2] Bakker P. A. H. M. Berendsen R. L. Doornbos R. F. Wintermans P. C. A. Pieterse C. M. J. (2013). The rhizosphere revisited: root microbiomics. Front. Plant Sci. 4:165. doi: 10.3389/fpls.2013.00165, PMID: 23755059 PMC3667247

[ref3] Bastian M. Heymann S. Jacomy M. (2009). Gephi: an open source software for exploring and manipulating networks. Proc. Int. AAAI Conf. Web Soc. Media 3, 361–362. doi: 10.1609/icwsm.v3i1.13937

[ref4] Bastida F. Eldridge D. J. García C. Kenny Png G. Bardgett R. D. Delgado-Baquerizo M. (2021). Soil microbial diversity-biomass relationships are driven by soil carbon content across global biomes. ISME J. 15, 2081–2091. doi: 10.1038/s41396-021-00906-0, PMID: 33564112 PMC8245509

[ref5] Bolyen E. Rideout J. R. Dillon M. R. Bokulich N. A. Abnet C. C. Al-Ghalith G. A.. (2019). Reproducible, interactive, scalable and extensible microbiome data science using QIIME 2. Nat. Biotechnol. 37, 852–857. doi: 10.1038/s41587-019-0209-9, PMID: 31341288 PMC7015180

[ref6] Bulgarelli D. Schlaeppi K. Spaepen S. van Themaat E. V. L. Schulze-Lefert P. (2013). Structure and functions of the bacterial microbiota of plants. Annu. Rev. Plant Biol. 64, 807–838. doi: 10.1146/annurev-arplant-050312-120106 23373698

[ref7] Cai Y. Luo Q. Sun M. Corke H. (2004). Antioxidant activity and phenolic compounds of 112 traditional Chinese medicinal plants associated with anticancer. Life Sci. 74, 2157–2184. doi: 10.1016/j.lfs.2003.09.047, PMID: 14969719 PMC7126989

[ref9] Chen M. Yang J. Xue C. Tu T. Su Z. Feng H.. (2024). Community composition of phytopathogenic fungi significantly influences ectomycorrhizal fungal communities during subtropical forest succession. Appl. Microbiol. Biotechnol. 108:99. doi: 10.1007/s00253-023-12992-5, PMID: 38204135 PMC10781812

[ref10] Chen S. Zhou Y. Chen Y. Gu J. (2018). Fastp: an ultra-fast all-in-one FASTQ preprocessor. Bioinformatics 34, i884–i890. doi: 10.1093/bioinformatics/bty560, PMID: 30423086 PMC6129281

[ref8] Chen Z. Du Z. Zhang Z. Wang G. Li J. (2023). Dynamic changes in soil organic carbon induced by long-term compost application under a wheat-maize double cropping system in North China. Sci. Total Environ. 913:169407. doi: 10.1016/j.scitotenv.2023.169407, PMID: 38123085

[ref11] Coleman-Derr D. Desgarennes D. Fonseca-Garcia C. Gross S. Clingenpeel S. Woyke T.. (2016). Plant compartment and biogeography affect microbiome composition in cultivated and native *Agave* species. New Phytol. 209, 798–811. doi: 10.1111/nph.13697, PMID: 26467257 PMC5057366

[ref12] Committee for the Pharmacopoeia of People’s Republic of China (2015). Pharmacopoeia of People’s Republic of China, part 1. Beijing: China Medical Science and Technology Press.

[ref13] de Cássia Mesquita da Cunha I. da Silva A. V. R. Boleta E. H. M. Pellegrinetti T. A. Zagatto L. F. G. Zagatto S. D. S. S.. (2024). The interplay between the inoculation of plant growth-promoting rhizobacteria and the rhizosphere microbiome and their impact on plant phenotype. Microbiol. Res. 283:127706. doi: 10.1016/j.micres.2024.127706, PMID: 38574431

[ref9001] Deng Y. Jiang Y. H. Yang Y. He Z. Luo F., and Zhou J. (2012). Molecular ecological network analyses. BMC Bioinf. 13:113. doi: 10.1186/1471-2105-13-113 PMC3428680 22646978

[ref14] Dennis P. G. Miller A. J. Hirsch P. R. (2010). Are root exudates more important than other sources of rhizodeposits in structuring rhizosphere bacterial communities? FEMS Microbiol. Ecol. 72, 313–327. doi: 10.1111/j.1574-6941.2010.00860.x 20370828

[ref15] Dhanabalan S. Muthusamy K. Iruthayasamy J. Kumaresan P. V. Ravikumar C. Kandasamy R.. (2024). Unleashing *Bacillus* species as versatile antagonists: harnessing the biocontrol potentials of the plant growth-promoting rhizobacteria to combat *Macrophomina phaseolina* infection in *Gloriosa superba*. Microbiol. Res. 283:127678. doi: 10.1016/j.micres.2024.127678, PMID: 38503218

[ref16] Douglas G. M. Maffei V. J. Zaneveld J. R. Yurgel S. N. Brown J. R. Taylor C. M.. (2020). PICRUSt2 for prediction of metagenome functions. Nat. Biotechnol. 38, 685–688. doi: 10.1038/s41587-020-0548-6, PMID: 32483366 PMC7365738

[ref17] Edwards J. Johnson C. Santos-Medellín C. Lurie E. Podishetty N. K. Bhatnagar S.. (2015). Structure, variation, and assembly of the root-associated microbiomes of rice. Proc. Natl. Acad. Sci. U.S.A. 112, E911–E920. doi: 10.1073/pnas.1414592112, PMID: 25605935 PMC4345613

[ref18] Feng Z. Xie X. Wu P. Chen M. Qin Y. Zhou Y.. (2023). Phenylalanine-mediated changes in the soil bacterial community promote nitrogen cycling and plant growth. Microbiol. Res. 275:127447. doi: 10.1016/j.micres.2023.127447, PMID: 37441843

[ref19] Frank A. C. Guzmán J. P. S. Shay J. E. (2017). Transmission of bacterial endophytes. Microorganisms 5:70. doi: 10.3390/microorganisms5040070, PMID: 29125552 PMC5748579

[ref20] Guo S. Tao C. Jousset A. Xiong W. Wang Z. Shen Z.. (2022). Trophic interactions between predatory protists and pathogen-suppressive bacteria impact plant health. ISME J. 16, 1932–1943. doi: 10.1038/s41396-022-01244-5, PMID: 35461357 PMC9296445

[ref21] Hannula S. E. Heinen R. Huberty M. Steinauer K. De Long J. R. Jongen R.. (2021). Persistence of plant-mediated microbial soil legacy effects in soil and inside roots. Nat. Commun. 12:5686. doi: 10.1038/s41467-021-25971-z, PMID: 34584090 PMC8478921

[ref22] Hardoim P. R. van Overbeek L. S. van Elsas J. D. (2008). Properties of bacterial endophytes and their proposed role in plant growth. Trends Microbiol. 16, 463–471. doi: 10.1016/j.tim.2008.07.008, PMID: 18789693

[ref23] Huang B. Chen Y. Pei Z. Jiang L. Zhang Y. Wang J.. (2023). Application of microbial organic fertilizers promotes the utilization of nutrients and restoration of microbial community structure and function in rhizosphere soils after dazomet fumigation. Front. Microbiol. 13:1122611. doi: 10.3389/fmicb.2022.1122611, PMID: 36741882 PMC9891460

[ref24] Jin S. Zhang B. Wu B. Han D. Hu Y. Ren C.. (2021). Decoupling livestock and crop production at the household level in China. Nat. Sustain. 4, 48–55. doi: 10.1038/s41893-020-00596-0

[ref25] Jogaiah S. Abdelrahman M. Tran L. S. P. Shin-Ichi I. (2013). Characterization of rhizosphere fungi that mediate resistance in tomato against bacterial wilt disease. J. Exp. Bot. 64, 3829–3842. doi: 10.1093/jxb/ert212, PMID: 23956415

[ref26] Kandasamy G. D. Kathirvel P. (2023). Insights into bacterial endophytic diversity and isolation with a focus on their potential applications—a review. Microbiol. Res. 266:127256. doi: 10.1016/j.micres.2022.127256, PMID: 36410317

[ref27] Klayman D. L. (1985). Qinghaosu (Artemisinin): an antimalarial drug from China. Science 228, 1049–1055. doi: 10.1126/science.3887571, PMID: 3887571

[ref28] Kong F. Ding Z. Zhang K. Duan W. Qin Y. Su Z.. (2020). Optimization of extraction flavonoids from *Exocarpium Citri Grandis* and evaluation its hypoglycemic and hypolipidemic activities. J. Ethnopharmacol. 262:113178. doi: 10.1016/j.jep.2020.113178, PMID: 32736047

[ref29] Larsen J. Jaramillo-López P. Nájera-Rincon M. Gonzaléz-Esquivel C. E. (2015). Biotic interactions in the rhizosphere in relation to plant and soil nutrient dynamics. J. Soil Sci. Plant Nutr. 15, 449–463. doi: 10.4067/s0718-95162015005000039

[ref30] Leite M. F. A. Dimitrov M. R. Freitas-Iório R. P. de Hollander M. Cipriano M. A. P. Andrade S. A. L.. (2021). Rearranging the sugarcane holobiont via plant growth-promoting bacteria and nitrogen input. Sci. Total Environ. 800:149493. doi: 10.1016/j.scitotenv.2021.149493, PMID: 34426366

[ref34] Ling N. Wang T. Kuzyakov Y. (2022). Rhizosphere bacteriome structure and functions. Nat. Commun. 13:836. doi: 10.1038/s41467-022-28448-9, PMID: 35149704 PMC8837802

[ref32] Li P. Liu M. H. Hu J. H. Su W. W. (2014). Systematic chemical profiling of *Citrus grandis* “Tomentosa” by ultra-fast liquid chromatography/diode-array detector/quadrupole time-of-flight tandem mass spectrometry. J. Pharm. Biomed. Anal. 90, 167–179. doi: 10.1016/j.jpba.2013.11.030, PMID: 24370611

[ref33] Li Q. Zhang D. Song Z. Ren L. Jin X. Fang W.. (2022). Organic fertilizer activates soil beneficial microorganisms to promote strawberry growth and soil health after fumigation. Environ. Pollut. 295:118653. doi: 10.1016/j.envpol.2021.118653, PMID: 34921948

[ref35] Liu Y. Tian Y. Yue L. Constantine U. Zhao X. Zhou Q.. (2021). Effectively controlling *Fusarium* root rot disease of *Angelica sinensis* and enhancing soil fertility with a novel attapulgite-coated biocontrol agent. Appl. Soil Ecol. 168:104121. doi: 10.1016/j.apsoil.2021.104121

[ref31] Li Y. Jin J. Li P. Wang Q. Xu L. Wei G.. (2023). Regional variations and plant compartments shape the community structures of the endophytic microbiome and secondary metabolites of *Astragalus mongholicus*. Ind. Crop. Prod. 192:116037. doi: 10.1016/j.indcrop.2022.116037

[ref36] Lundberg D. S. Lebeis S. L. Paredes S. H. Yourstone S. Gehring J. Malfatti S.. (2012). Defining the core *Arabidopsis thaliana* root microbiome. Nature 488, 86–90. doi: 10.1038/nature11237, PMID: 22859206 PMC4074413

[ref37] Lutzoni F. Nowak M. D. Alfaro M. E. Reeb V. Miadlikowska J. Krug M.. (2018). Contemporaneous radiations of fungi and plants linked to symbiosis. Nat. Commun. 9:5451. doi: 10.1038/s41467-018-07849-9, PMID: 30575731 PMC6303338

[ref39] Magoč T. Salzberg S. L. (2011). FLASH: fast length adjustment of short reads to improve genome assemblies. Bioinformatics 27, 2957–2963. doi: 10.1093/bioinformatics/btr507, PMID: 21903629 PMC3198573

[ref38] Ma H. Pineda A. Hannula S. E. Kielak A. M. Setyarini S. N. Bezemer T. M. (2020). Steering root microbiomes of a commercial horticultural crop with plant-soil feedbacks. Appl. Soil Ecol. 150:103468. doi: 10.1016/j.apsoil.2019.103468

[ref40] Obidola S. M. Iro I. I. Rebecca Z. A. (2019). Influence of organic manure and inorganic fertilizer on the growth, yield and phytochemical constituents of cabbage (*Brassica oleracea*). Asian J. Agric. Res. 4, 1–9. doi: 10.9734/ajahr/2019/v4i130012

[ref41] Olesen J. M. Bascompte J. Dupont Y. L. Jordano P. (2007). The modularity of pollination networks. Proc. Natl. Acad. Sci. U.S.A. 104, 19891–19896. doi: 10.1073/pnas.0706375104, PMID: 18056808 PMC2148393

[ref42] Pacheco A. C. Feba L. G. T. Serra E. G. Takata W. H. S. Gorni P. H. Yoshida C. H. P. (2021). The use of animal manure in the organic cultivation of *Passiflora incarnata* L. increases the content of phenolic compounds in the leaf and the antioxidant activity of the plant. Org. Agric. 11, 567–575. doi: 10.1007/s13165-021-00361-3

[ref43] Philippot L. Raaijmakers J. M. Lemanceau P. van der Putten W. H. (2013). Going back to the roots: the microbial ecology of the rhizosphere. In. Nat. Rev. Microbiol. 11, 789–799. doi: 10.1038/nrmicro3109 24056930

[ref44] Pu R. Wang P. Guo L. Li M. Cui X. Wang C.. (2022). The remediation effects of microbial organic fertilizer on soil microorganisms after chloropicrin fumigation. Ecotoxicol. Environ. Saf. 231:113188. doi: 10.1016/j.ecoenv.2022.113188, PMID: 35051756

[ref45] Qian P. Schoenau J. J. (2002). Availability of nitrogen in solid manure amendments with different C:N ratios. Can. J. Soil Sci. 82, 219–225. doi: 10.4141/S01-018

[ref46] Raaijmakers J. M. Paulitz T. C. Steinberg C. Alabouvette C. Moënne-Loccoz Y. (2009). The rhizosphere: a playground and battlefield for soilborne pathogens and beneficial microorganisms. Plant Soil 321, 341–361. doi: 10.1007/s11104-008-9568-6

[ref47] Ruan Z. Chen K. Cao W. Meng L. Yang B. Xu M.. (2024). Engineering natural microbiomes toward enhanced bioremediation by microbiome modeling. Nat. Commun. 15:4694. doi: 10.1038/s41467-024-49098-z, PMID: 38824157 PMC11144243

[ref48] Shaji H. Chandran V. Mathew L. (2021). “Organic fertilizers as a route to controlled release of nutrients” in Controlled release fertilizers for sustainable agriculture (Cambridge, MA: Academic Press).

[ref49] Shu X. He J. Zhou Z. Xia L. Hu Y. Zhang Y.. (2022). Organic amendments enhance soil microbial diversity, microbial functionality and crop yields: a meta-analysis. Sci. Total Environ. 829:154627. doi: 10.1016/j.scitotenv.2022.154627, PMID: 35306065

[ref50] Spanoghe J. Grunert O. Wambacq E. Sakarika M. Papini G. Alloul A.. (2020). Storage, fertilization and cost properties highlight the potential of dried microbial biomass as organic fertilizer. Microb. Biotechnol. 13, 1377–1389. doi: 10.1111/1751-7915.13554, PMID: 32180337 PMC7415357

[ref51] Sun R. Yi Z. Fu Y. Liu H. (2022). Dynamic changes in rhizosphere fungi in different developmental stages of wheat in a confined and isolated environment. Appl. Microbiol. Biotechnol. 106, 441–453. doi: 10.1007/s00253-021-11698-w, PMID: 34870738

[ref52] Trivedi P. Leach J. E. Tringe S. G. Sa T. Singh B. K. (2020). Plant-microbiome interactions: from community assembly to plant health. Nat. Rev. Microbiol. 18, 607–621. doi: 10.1038/s41579-020-0412-1 32788714

[ref53] Verma H. Patra R. K. Sethi D. Pattanayak S. K. (2022). Isolation and characterization of native Rhizobium from root nodules of raikia french bean growing area of Odisha. Indian J. Biochem. Biophys. 59, 918–926. doi: 10.56042/ijbb.v59i9.61519

[ref55] Wang B. Huang Y. Liu W. Chen S. Zhu J. Belzile N.. (2021). Returning excrement from livestock, poultry, and humans to farmland as nutrient resources for crop growth: assessment of rural China. Process Saf. Environ. Prot. 146, 412–423. doi: 10.1016/j.psep.2020.09.001

[ref54] Wang Y. Duan S. Xu J. Long Y. Zhang S. Li S.. (2023). Comparison of the colonization ability of Burkholderia strain B23 in the citrus rhizoplane and rhizosphere and assessment of the underlying mechanisms using full-length 16S rDNA amplicon and metatranscriptomic analyses. Microb. Biotechnol. 16, 1657–1670. doi: 10.1111/1751-7915.14248, PMID: 36946260 PMC10364312

[ref56] Wang Z. Yin Y. Wang Y. Tian X. Ying H. Zhang Q.. (2022). Integrating crop redistribution and improved management towards meeting China’s food demand with lower environmental costs. Nat. Food 3, 1031–1039. doi: 10.1038/s43016-022-00646-0, PMID: 37118293

[ref57] Wei Z. Gu Y. Friman V. P. Kowalchuk G. A. Xu Y. Shen Q.. (2019). Initial soil microbiome composition and functioning predetermine future plant health. Sci. Adv. 5:eaaw0759. doi: 10.1126/sciadv.aaw0759, PMID: 31579818 PMC6760924

[ref59] Wen T. Yuan J. He X. Lin Y. Huang Q. Shen Q. (2020). Enrichment of beneficial cucumber rhizosphere microbes mediated by organic acid secretion. Hortic. Res. 7:154. doi: 10.1038/s41438-020-00380-3, PMID: 33082961 PMC7527982

[ref58] Wen Y. C. Li H. Y. Lin Z. A. Zhao B. Q. Sun Z. B. Yuan L.. (2020). Long-term fertilization alters soil properties and fungal community composition in fluvo-aquic soil of the North China Plain. Sci. Rep. 10:7198. doi: 10.1038/s41598-020-64227-6, PMID: 32350351 PMC7190697

[ref60] Xiao J. Zhang J. Yuan H. Xie X. Gao Y. Lu Y.. (2024). Long-term application of legume green manure improves rhizosphere soil bacterial stability and reduces bulk soil bacterial stability in rice. Eur. J. Soil Biol. 122:103652. doi: 10.1016/J.EJSOBI.2024.103652

[ref63] Xun W. Yan R. Ren Y. Jin D. Xiong W. Zhang G.. (2018). Grazing-induced microbiome alterations drive soil organic carbon turnover and productivity in meadow steppe. Microbiome 6:170. doi: 10.1186/s40168-018-0544-y, PMID: 30236158 PMC6149009

[ref61] Xu P. Li G. Zheng Y. Fung J. C. H. Chen A. Zeng Z.. (2024). Fertilizer management for global ammonia emission reduction. Nature 626, 792–798. doi: 10.1038/s41586-024-07020-z, PMID: 38297125

[ref62] Xu Q. Ling N. Chen H. Duan Y. Wang S. Shen Q.. (2020). Long-term chemical-only fertilization induces a diversity decline and deep selection on the soil bacteria. mSystems 5:e00337. doi: 10.1128/msystems.00337-20, PMID: 32665327 PMC7363003

[ref64] Yang J. Lin Y. (2019). Spatiotemporal evolution and driving factors of fertilizer reduction control in Zhejiang Province. Sci. Total Environ. 660, 650–659. doi: 10.1016/j.scitotenv.2018.12.420, PMID: 30641394

[ref67] Zhang J. Ye L. Chang J. Wang E. Wang C. Zhang H.. (2024). Straw soil conditioner modulates key soil microbes and nutrient dynamics across different maize developmental stages. Microorganisms 12:295. doi: 10.3390/microorganisms12020295, PMID: 38399698 PMC10893213

[ref65] Zhang S. Li X. Chen K. Shi J. Wang Y. Luo P.. (2022). Long-term fertilization altered microbial community structure in an aeolian sandy soil in Northeast China. Front. Microbiol. 13:979759. doi: 10.3389/fmicb.2022.979759, PMID: 36160213 PMC9490088

[ref66] Zhang W. J. Wang X. J. Xu M. G. Huang S. M. Liu H. Peng C. (2010). Soil organic carbon dynamics under long-term fertilizations in arable land of northern China. Biogeosciences 7, 409–425. doi: 10.5194/bg-7-409-2010

[ref68] Zhao Z. Ma Y. Zhang A. Chen Y. Zheng Z. Zheng W.. (2023). Response of apple orchard bacteria co-occurrence network pattern to long-term organic fertilizer input. Appl. Soil Ecol. 191:105035. doi: 10.1016/j.apsoil.2023.105035

[ref69] Zhou X. Wang J. Liu F. Liang J. Zhao P. Tsui C. K. M.. (2022). Cross-kingdom synthetic microbiota supports tomato suppression of *Fusarium* wilt disease. Nat. Commun. 13:7890. doi: 10.1038/s41467-022-35452-6, PMID: 36550095 PMC9780251

[ref70] Zhu Z. Zhang X. Dong H. Wang S. Reis S. Li Y.. (2022). Integrated livestock sector nitrogen pollution abatement measures could generate net benefits for human and ecosystem health in China. Nat. Food 3, 161–168. doi: 10.1038/s43016-022-00462-6, PMID: 37117962

[ref71] Zuo J. Zu M. Liu L. Song X. Yuan Y. (2021). Composition and diversity of bacterial communities in the rhizosphere of the Chinese medicinal herb Dendrobium. BMC Plant Biol. 21:127. doi: 10.1186/s12870-021-02893-y, PMID: 33663379 PMC7931511

