# Supplementary material for: Adaptation of rhizobacterial and endophytic communities in Citrus Grandis Exocarpium to long-term organic and chemical fertilization
Source: Front Microbiol. 2024 Oct 21;15:1461821. doi: 10.3389/fmicb.2024.1461821 (PMC11532108; doi:10.3389/fmicb.2024.1461821)
Supplement: Supplementary file 1 [file Data_Sheet_1.PDF]

Supplementary materials for

Frontiers in Microbiology

Adaptation of rhizobacterial and endophytic communities in *Citrus Grandis Exocarpium* to long-term organic and chemical fertilization

Deyang Zhou[a#], Kaiqing Yang[a#], Yinhui Zhang[a], Cancan Liu[a], Ye He[a], Jialin Tan[a], Zhepu Ruan[a]*, Rongliang Qiu[ab]

[a] Guangdong Laboratory for Lingnan Modern Agriculture, Guangdong Provincial Key Laboratory of Agricultural & Rural Pollution Abatement and Environmental Safety, College of Natural Resources and Environment, South China Agricultural University, Guangzhou 510642, China.

[b] School of Environmental Science and Engineering, Sun Yat-sen University, Guangzhou 510006, China.

[#]Deyang Zhou and Kaiqing Yang contribute equally to this work.

*Corresponding author.

E-mail address: ruanzhepu@scau.edu.cn (Z. Ruan)

Supplementary Methods

The LC-MS/MS analysis of sample was conducted on a Thermo UHPLC-Q Exactive HF-X system equipped with an ACQUITY HSS T3 column (100 mm × 2.1 mm i.d., 1.8 µm; Waters, USA) at Majorbio Bio-Pharm Technology Co. Ltd. (Shanghai, China). The mobile phases consisted of 0.1% formic acid in water:acetonitrile (95:5, v/v) (solvent A) and 0.1% formic acid in acetonitrile:isopropanol: water (47.5:47.5, v/v) (solvent B). Positive ion mode separation gradient: 0-3 min, mobile phase B was increased from 0% to 20%; 3-4.5 min, mobile phase B was increased from 20% to 35%; 4.5-5 min, mobile phase B was increased from 35% to 100%; 5-6.3 min, mobile phase B was maintained at 100%; 6.3-6.4 min, mobile phase B was decreased from 100% to 0%; 6.4-8 min , mobile phase B was maintained at 0%. Separation gradient in negative ion mode: 0-1.5 min, mobile phase B rises from 0 to 5%; 1.5-2 min, mobile phase B rises from 5% to 10%; 2-4.5 min, mobile phase B rises from 10% to 30%; 4.5-5 min, mobile phase B rises from 30% to 100%; 5-6.3 min, mobile phase B linearly maintains 100%; 6.3-6.4 min, the mobile phase B decreased from 100% to 0%; 6.4-8 min, the mobile phase B was linearly maintained at 0%. The flow rate was 0.40 mL/min and the column temperature was 40℃.

MS conditions:

The mass spectrometric data were collected using a Thermo UHPLC-Q Exactive HF-X Mass Spectrometer equipped with an electrospray ionization (ESI) source operating in positive mode and negative mode. The optimal conditions were set as followed: source temperature at 425℃ ; sheath gas flow rate at 50 arb; Aux gas flow rate at 13 arb; ion-spray voltage floating (ISVF) at -3500V in negative mode and 3500V in positive mode, respectively; Normalized collision energy , 20-40-60V rolling for MS/MS. Full MS resolution was 60000, and MS/MS resolution was 7500. Data acquisition was performed with the Data Dependent Acquisition (DDA) mode. The detection was carried out over a mass range of 70-1050 m/z.

The pretreatment of LC/MS raw data was performed by Progenesis QI (Waters Corporation, Milford, USA) software. Internal standard peaks, as well as any known false positive peaks (including noise, column bleed, and derivatized reagent peaks), were removed from the data matrix, deredundant and peak pooled. At the same time, the metabolites were identified by searching database, and the main databases were the HMDB (http://www.hmdb.ca/), Metlin (https://metlin.scripps.edu/) and Majorbio Database.

The data matrix was pre-processed, as follows: At least 80% of the metabolic features detected in any set of samples were retained. After filtering, for specific samples with metabolite levels below the lower limit of quantification, the minimum metabolite value was estimated and each metabolic signature was normalized to the sum. To reduce the errors caused by sample preparation and instrument instability, the response intensities of the sample mass spectrometry peaks were normalized using the sum normalization method, to obtain the normalized data matrix. Meanwhile, the variables of QC samples with relative standard deviation (RSD) > 30% were excluded and log10 logarithmicized, to obtain the final data matrix for subsequent analysis.

Supplementary Figures

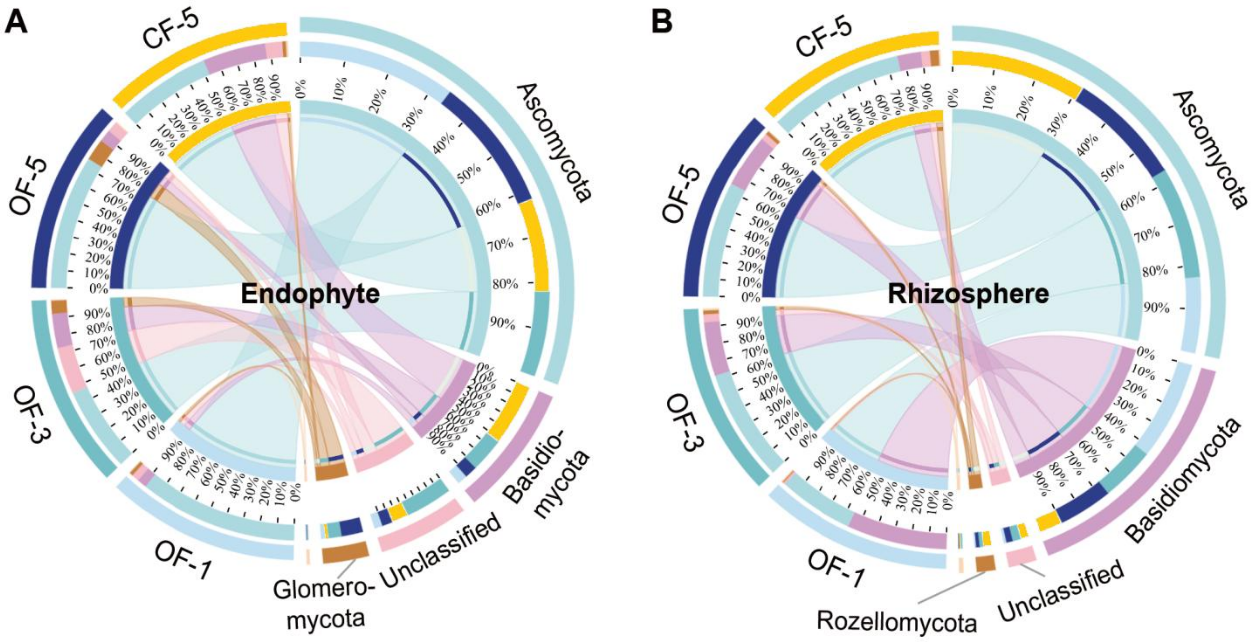

Supplementary Fig. S1. Circos diagram of species composition of fungi at phylum level. A, endophyte; B. rhizosphere fungi. OF-1, one-year tree treated by organic fertilizer; OF-3, three-year tree treated by organic fertilizer; OF-5, five-year tree treated by organic fertilizer; CF-5, five-year tree treated by chemical fertilizer.

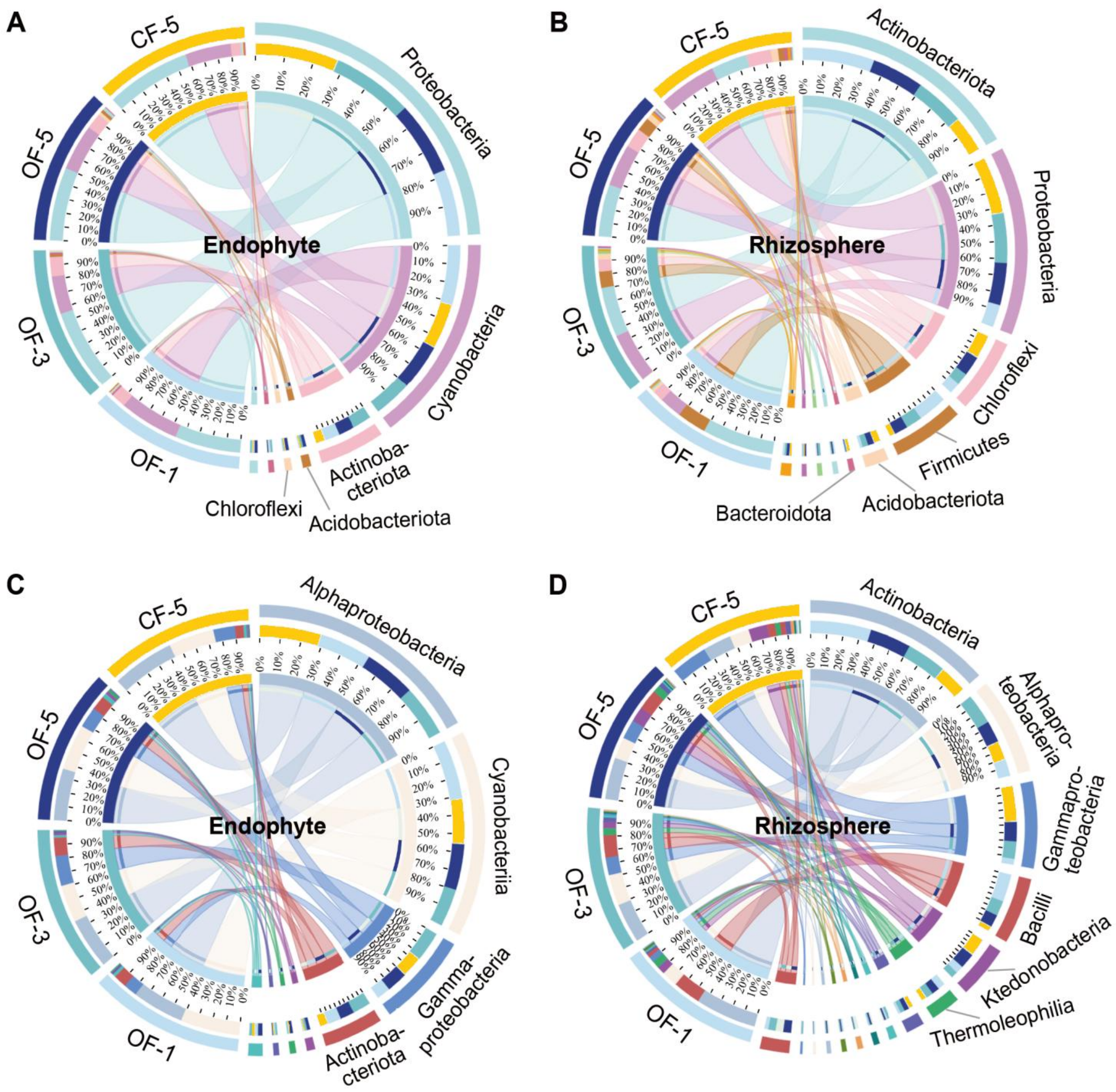

Supplementary Fig. S2. Circos diagram of species composition of bacteria. A, endophyte at phylum level; B, rhizosphere bacteria at phylum level; C, endophyte at class level; D, rhizosphere bacteria at class level. OF-1, one-year tree treated by organic fertilizer; OF-3, three-year tree treated by organic fertilizer; OF-5, five-year tree treated by organic fertilizer; CF-5, five-year tree treated by chemical fertilizer.

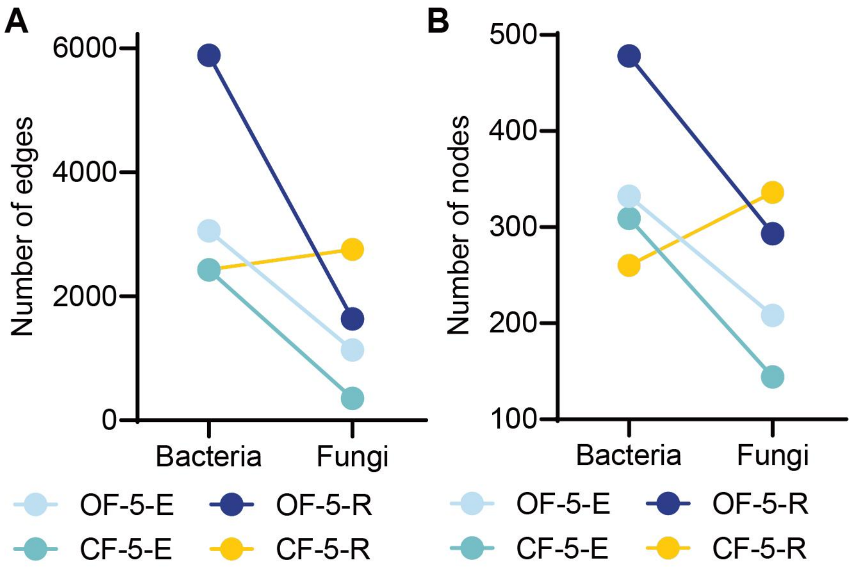

Supplementary Fig. S3. Correlation network properties of bacterial and fungal groups. A, Number of edges; B, number of nodes. OF-5, five-year tree treated by organic fertilizer; CF-5, five-year tree treated by chemical fertilizer. E, Endophyte; R, rhizosphere.

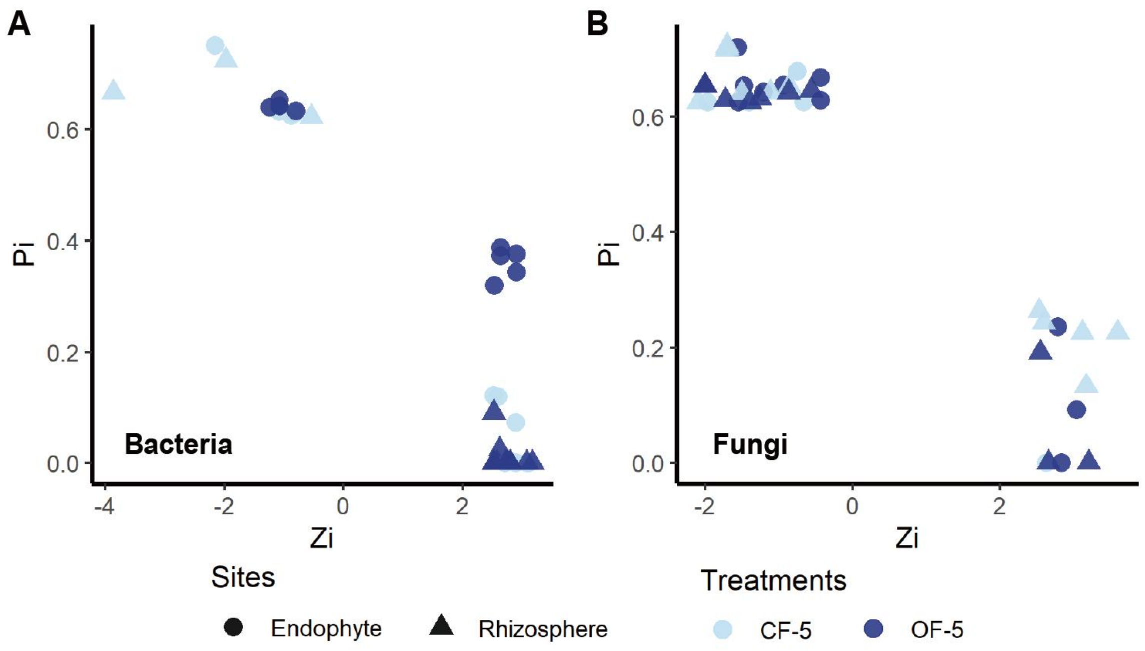

Supplementary Fig. S4. Zi-Pi value of the nodes of the correlation network of bacterial and fungal groups. A, Bacteria; B, fungi. OF-5, five-year tree treated by organic fertilizer; CF-5, five-year tree treated by chemical fertilizer.

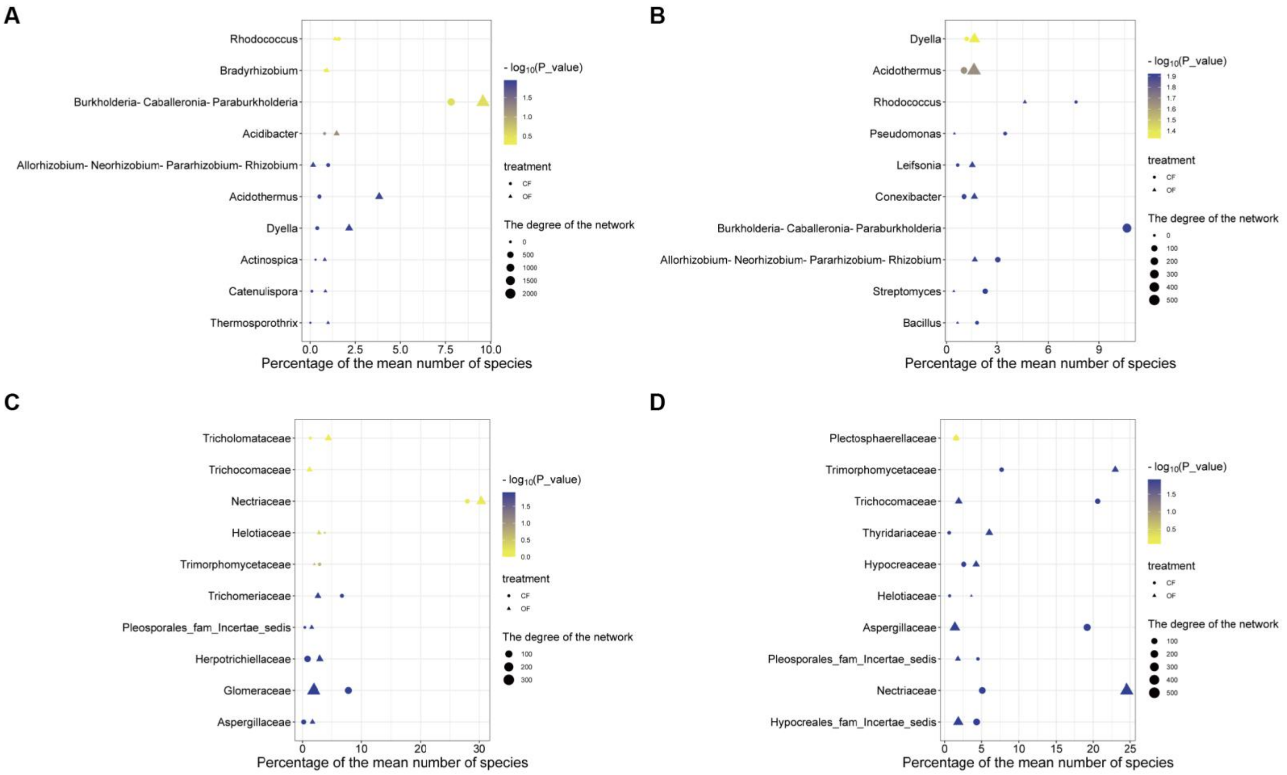

Supplementary Fig. S5. Analysis of top ten species abundance in the co-occurrence networks of different treatments. A, in Bacteria-Endophyte groups at genus level; B, Bacteria-Rhizosphere at genus level; C, Fungi-Endophyte at family level; D, Fungi-Rhizosphere at family level. OF, organic fertilizer; CF, chemical fertilizer.

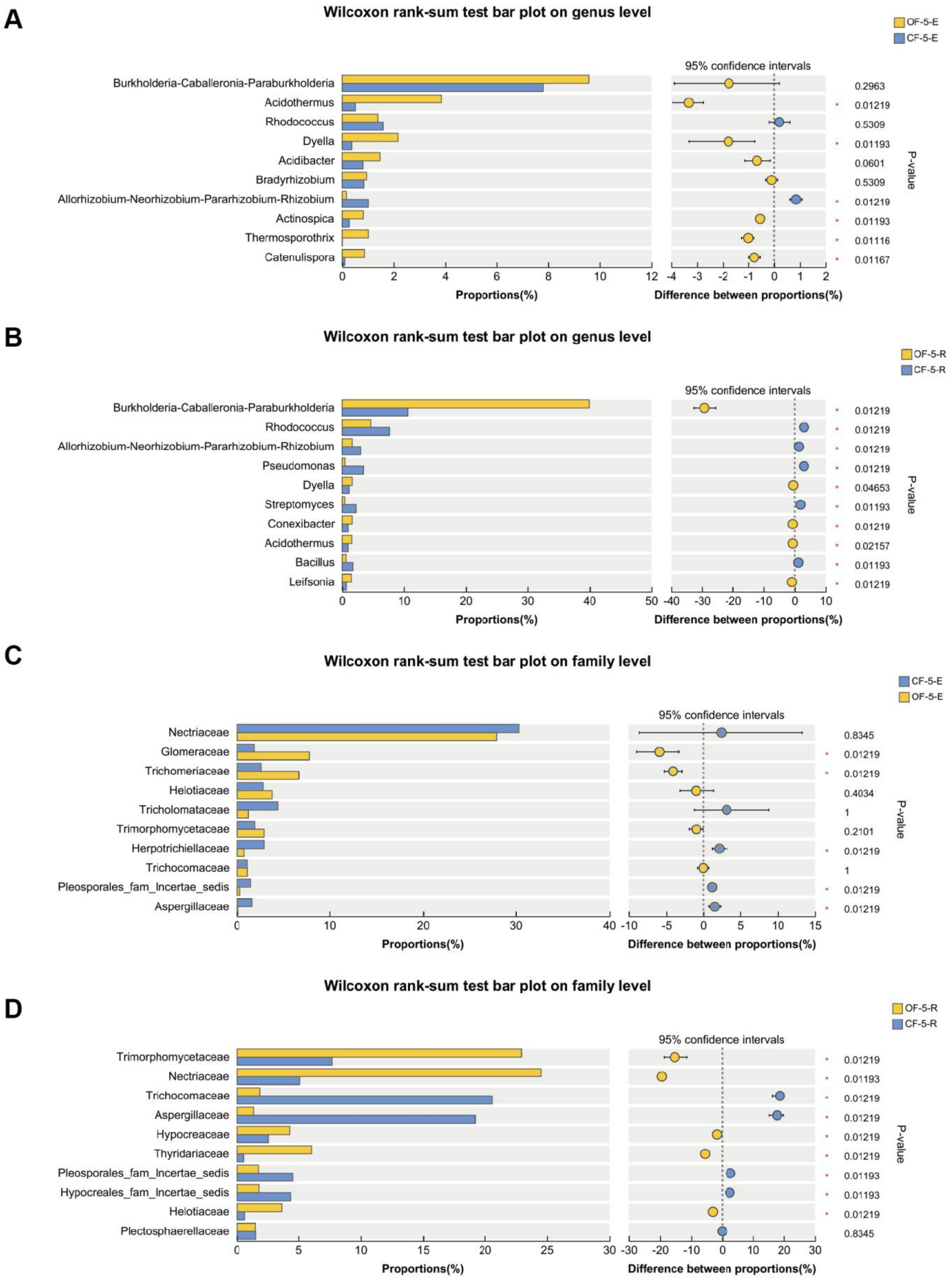

Supplementary Fig. S6. Analysis of species differences between the chemical fertilizer treatment and the organic fertilizer treatment at the fifth year. A, Bacteria-Endophyte; B, Bacteria-Rhizosphere; C, Fungi-Endophyte; D, Fungi-Rhizosphere. *, $P < 0.05$.

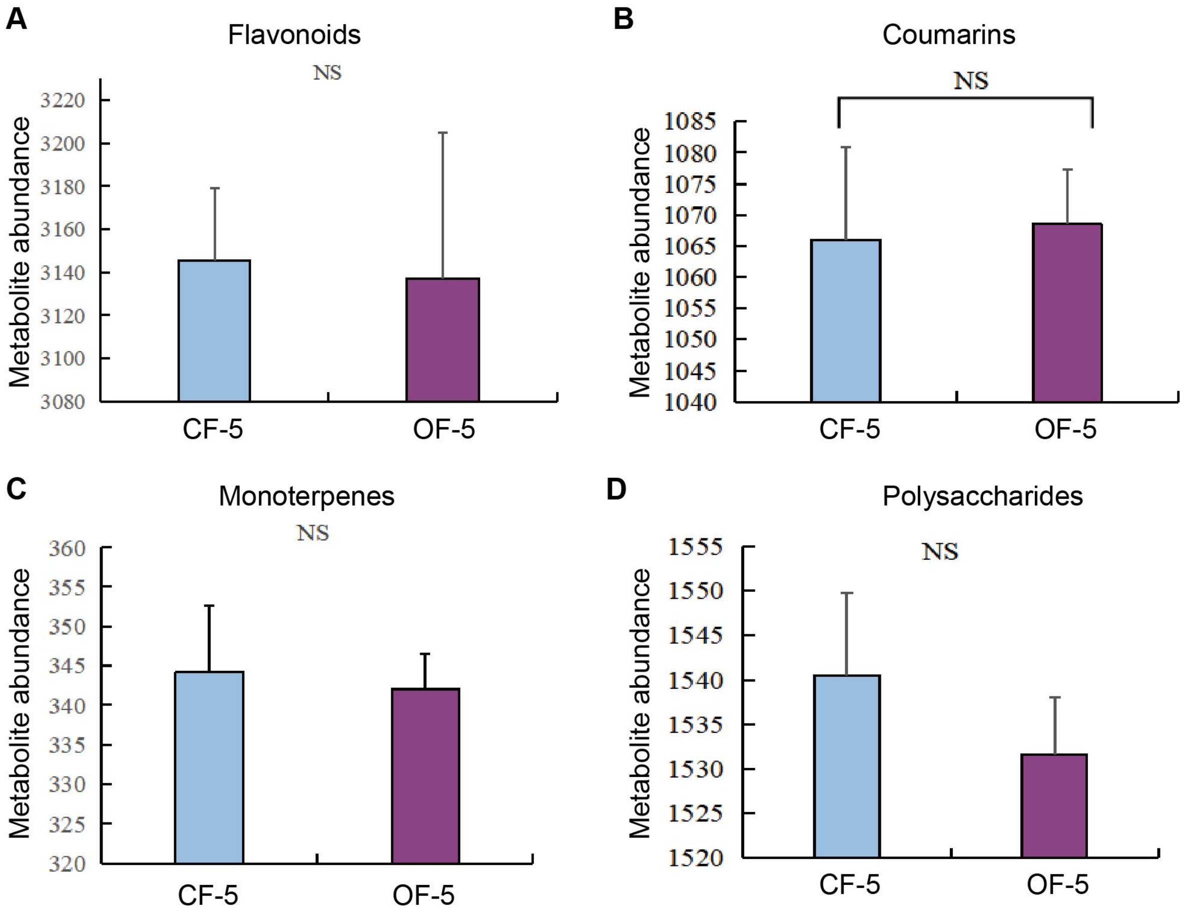

Supplementary Fig. S7. Analysis of metabolites content of flavonoids, polysaccharides, monoterpenes, and coumarinsof Huajuhong. OF-5, five-year tree treated by organic fertilizer; CF-5, five-year tree treated by chemical fertilizer.

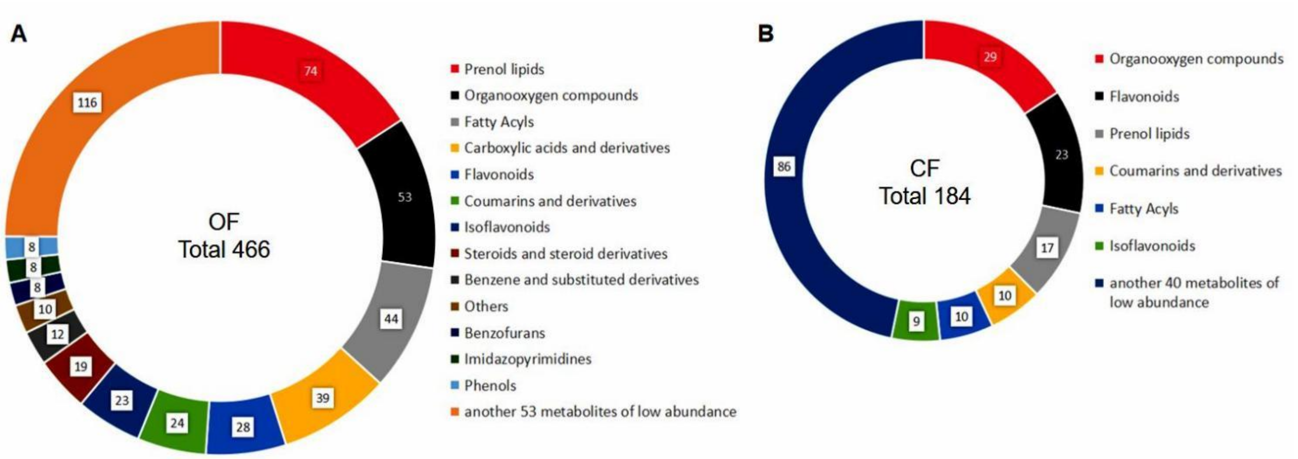

Supplementary Fig. S8. Information of substances significantly upregulated in the OF or CF treated Huajuhong peel. A, OF treated Huajuhong peel; B, CF treated Huajuhong peel. OF, organic fertilizer; CF, chemical fertilizer.

Supplementary Tables

Supplementary Table S1. Basic chemical properties of soil. TOC, total organic carbon; TN, total nitrogen; DOC, dissolved organic carbon.

Basic chemical properties of soil

	OF-1	OF-3	OF-5	CF-5
pH value	4.95	4.33	4.30	4.22
N-NH_4^+ (mg/kg)	65.1	14.9	19.5	31
N-NO_3^- (mg/kg)	1.43	1.38	0.98	1.79
Available P (mg/kg)	70.6	838	22.6	141
TOC (g/kg)	1.23	4.09	4.88	8.69
TN (g/kg)	0.434	0.472	0.520	0.754
DOC (mg/kg)	44.64	74.19	69.86	101
Available N (mg/kg)	46.9	50.3	54.6	77.3
Available K (mg/kg)	98.7	94.3	79.4	106

Supplementary Table S2. Information of the top 10 species of endophytic bacteria abundance in each group. Green background, probiotics; red background, plant pathogen.

OF-1-E			OF-3-E			OF-5-E			CF-5-E		
ASV ID	Average number	Genus	ASV ID	Average number	Genus	ASV ID	Average number	Genus	ASV ID	Average number	Genus
ASV2	1945	Rhodococcus	ASV2	1748	Rhodococcus	ASV2	612	Rhodococcus	ASV6	1536	Burkholderia-Caballeronia-Paraburkholderia
ASV14	257	Bradyrhizobium	ASV6	1198	Burkholderia-Caballeronia-Paraburkholderia	ASV7	522	Burkholderia-Caballeronia-Paraburkholderia	ASV8	904	Burkholderia-Caballeronia-Paraburkholderia
ASV11	42	Allorhizobium-Neorhizobium-Pararhizobium-Rhizobium	ASV11	933	Allorhizobium-Neorhizobium-Pararhizobium-Rhizobium	ASV6	471	Burkholderia-Caballeronia-Paraburkholderia	ASV2	792	Rhodococcus
ASV200	21	Bacillus	ASV217	630	Burkholderia-Caballeronia-Paraburkholderia	ASV14	427	Bradyrhizobium	ASV4	408	Burkholderia-Caballeronia-Paraburkholderia
ASV528	10	Bacillus	ASV2461	522	Phyllobacterium	ASV224	392	Dyella	ASV24	375	Enterobacter
ASV369	1	Pantoea	ASV122	471	Dyella	ASV79	339	Catenulispora	ASV34	329	Burkholderia-Caballeronia-Paraburkholderia
ASV381	1	Pantoea	ASV82	464	Burkholderia-Caballeronia-Paraburkholderia	ASV5	324	Burkholderia-Caballeronia-Paraburkholderia	ASV7	314	Burkholderia-Caballeronia-Paraburkholderia
ASV103	1	Pseudarthrobacter	ASV8079	403	Kutzneria	ASV96	320	Myrothecium	ASV11	313	Allorhizobium-Neorhizobium-Pararhizobium-Rhizobium
ASV7319	0	Paenarthrobacter	ASV34	275	Burkholderia-Caballeronia-Paraburkholderia	ASV9	269	Burkholderia-Caballeronia-Paraburkholderia	ASV2470	273	Stenotrophomonas

ASV775	0	Paenarthrobacter	ASV144	250	Bradyrhizobium	ASV34	265	Burkholderia-Caballeronia-Paraburkholderia	ASV14	267	Bradyrhizobium
Sum (probiotics)	2278		Sum (probiotics)	6895		Sum (probiotics)	3621		Sum (probiotics)	5238	

Supplementary Table S3. Information of the top 10 species of endophytic fungi abundance in each group. Green background, probiotics; red background, plant pathogen.

OF-1-E			OF-3-E			OF-5-E			CF-5-E		
ASV ID	Average number	Genus	ASV ID	Average number	Genus	ASV ID	Average number	Genus	ASV ID	Average number	Genus
ASV1	18802	Fusarium	ASV1	14403	Fusarium	ASV1	11339	Fusarium	ASV1	16299	Fusarium
ASV11	10697	Neocosmospora	ASV98	3217	Talaromyces	ASV20	8851	Neocosmospora	ASV133	2542	Marasmiellus
ASV14	3643	Neocosmospora	ASV20	1954	Neocosmospora	ASV136	3342	Knufia	ASV8	1545	Scytalidium
ASV3	3226	Trichoderma	ASV136	1835	Knufia	ASV8	2950	Scytalidium	ASV136	1121	Knufia
ASV9	2326	Trichoderma	ASV449	1221	Rhizophagus	ASV23	2311	Saitozyma	ASV23	1048	Saitozyma
ASV13	1981	Trichoderma	ASV26	868	Trichoderma	ASV154	1863	Knufia	ASV73	804	Pyrenochaetopsis
ASV5	1423	Neocosmospora	ASV451	778	Rhizophagus	ASV11	1220	Neocosmospora	ASV12	624	Talaromyces
ASV12	1398	Talaromyces	ASV453	743	Rhizophagus	ASV12	801	Talaromyces	ASV6	527	Penicillium
ASV6	1338	Penicillium	ASV454	684	Arnium	ASV204	540	Trichoderma	ASV139	463	Cladophialophora
ASV8	1335	Scytalidium	ASV27	603	Cladosporium	ASV195	534	Roussoella	ASV11	437	Neocosmospora
Sum (probiotics)	0		Sum (probiotics)	8477		Sum (probiotics)	8050		Sum (probiotics)	5173	

Supplementary Table S4. Information of the top 10 species of rhizosphere bacteria abundance in each group. Green background, probiotics; red background, plant pathogen.

OF-1-R			OF-3-R			OF-5-R			CF-5-R		
ASV ID	Average number	Genus	ASV ID	Average number	Genus	ASV ID	Average number	Genus	ASV ID	Average number	Genus
ASV2	20791	Rhodococcus	ASV2	13636	Rhodococcus	ASV2	3111	Rhodococcus	ASV2	6655	Rhodococcus
ASV200	9539	Bacillus	ASV11	8416	Allorhizobium-Neorhizobium-Pararhizobium-Rhizobium	ASV11	955	Allorhizobium-Neorhizobium-Pararhizobium-Rhizobium	ASV11	2187	Allorhizobium-Neorhizobium-Pararhizobium-Rhizobium
ASV528	5126	Bacillus	ASV6	1096	Burkholderia-Caballeronia-Paraburkholderia	ASV14	517	Bradyrhizobium	ASV14	510	Bradyrhizobium
ASV11	3162	Allorhizobium-Neorhizobium-Pararhizobium-Rhizobium	ASV217	925	Burkholderia-Caballeronia-Paraburkholderia	ASV46	195	Curtobacterium	ASV381	296	Pantoea
ASV10	1344	Leifsonia	ASV3072	860	Kroppenstedtia	ASV103	66	Pseudarthrobacter	ASV46	169	Curtobacterium
ASV102	1070	Phaeosphaeriopsis	ASV200	718	Bacillus	ASV200	66	Bacillus	ASV200	99	Bacillus
ASV14	825	Bradyrhizobium	ASV14	717	Bradyrhizobium	ASV381	39	Pantoea	ASV369	73	Pantoea
ASV91	767	Conexibacter	ASV1067	613	Allorhizobium-Neorhizobium-Pararhizobium-Rhizobium	ASV528	18	Bacillus	ASV103	60	Pseudarthrobacter
ASV65	513	Leifsonia	ASV82	580	Burkholderia-Caballeronia-Paraburkholderia	ASV7319	13	Paenarthrobacter	ASV528	57	Bacillus
ASV103	496	Pseudarthrobacter	ASV820	567	Rhodococcus	ASV369	12	Pantoea	ASV3064	53	Microbacterium
Sum (probiotics)	42563		Sum (probiotics)	27268		Sum (probiotics)	4990		Sum (probiotics)	10160	

Supplementary Table S5. Information of the top 10 species of rhizosphere fungi abundance in each group. Green background, probiotics; red background, plant pathogen.

OF-1-R			OF-3-R			OF-5-R			CF-5-R		
ASV ID	Average number	Genus	ASV ID	Average number	Genus	ASV ID	Average number	Genus	ASV ID	Average number	Genus
ASV23	44705	Saitozyma	ASV23	23441	Saitozyma	ASV23	23570	Saitozyma	ASV893	15682	Talaromyces
ASV172	6941	Saitozyma	ASV1	8425	Fusarium	ASV1	10758	Fusarium	ASV23	7466	Saitozyma
ASV1	5109	Fusarium	ASV73	5253	Pyrenochaetopsis	ASV20	7727	Neocosmospora	ASV73	4533	Pyrenochaetopsis
ASV45	4155	Penicillium	ASV308	4716	Eleutherascus	ASV195	5710	Roussoella	ASV12	3287	Talaromyces
ASV48	1864	Cladophialophora	ASV20	3710	Neocosmospora	ASV8	3916	Scytalidium	ASV1	2679	Fusarium
ASV12	1773	Talaromyces	ASV98	3395	Talaromyces	ASV204	2371	Trichoderma	ASV894	1721	Chaetomium
ASV1783	1743	Trichosporon	ASV1392	2159	Leucothecium	ASV347	2182	Papiliotrema	ASV234	1711	Acremonium
ASV16	1554	Trichoderma	ASV369	1726	Sarocladium	ASV11	2090	Neocosmospora	ASV98	1498	Talaromyces
ASV11	1233	Neocosmospora	ASV82	1292	Talaromyces	ASV73	1822	Pyrenochaetopsis	ASV308	1228	Eleutherascus
ASV308	1030	Eleutherascus	ASV1387	1054	Sagenomella	ASV136	1580	Knufia	ASV162	1213	Gibellulopsis
Sum (probiotics)	56283		Sum (probiotics)	29212		Sum (probiotics)	33042		Sum (probiotics)	13339	
